# Classification, replication, and transcription of *Nidovirales*

**DOI:** 10.3389/fmicb.2023.1291761

**Published:** 2024-01-24

**Authors:** Ying Liao, Huan Wang, Huiyu Liao, Yingjie Sun, Lei Tan, Cuiping Song, Xusheng Qiu, Chan Ding

**Affiliations:** ^1^Department of Avian Diseases, Shanghai Veterinary Research Institute, Chinese Academy of Agricultural Sciences, Shanghai, China; ^2^Jiangsu Co-Innovation Center for Prevention and Control of Important Animal Infectious Diseases and Zoonoses, Yangzhou, China

**Keywords:** *Nidovirales*, replication-transcription complex, dis-continuous RNA synthesis, transcription regulatory sequence, replicase, host factor

## Abstract

*Nidovirales* is one order of RNA virus, with the largest single-stranded positive sense RNA genome enwrapped with membrane envelope. It comprises four families (*Arterividae, Mesoniviridae, Roniviridae, and Coronaviridae*) and has been circulating in humans and animals for almost one century, posing great threat to livestock and poultry，as well as to public health. *Nidovirales* shares similar life cycle: attachment to cell surface, entry, primary translation of replicases, viral RNA replication in cytoplasm, translation of viral proteins, virion assembly, budding, and release. The viral RNA synthesis is the critical step during infection, including genomic RNA (gRNA) replication and subgenomic mRNAs (sg mRNAs) transcription. gRNA replication requires the synthesis of a negative sense full-length RNA intermediate, while the sg mRNAs transcription involves the synthesis of a nested set of negative sense subgenomic intermediates by a discontinuous strategy. This RNA synthesis process is mediated by the viral replication/transcription complex (RTC), which consists of several enzymatic replicases derived from the polyprotein 1a and polyprotein 1ab and several cellular proteins. These replicases and host factors represent the optimal potential therapeutic targets. Hereby, we summarize the *Nidovirales* classification, associated diseases, “replication organelle,” replication and transcription mechanisms, as well as related regulatory factors.

## 1 Introduction

Named after the Latin word “nidus” (meaning nest), *Nidovirales* refers to an order of viruses which produce a 3′ co-terminal nested set of sg mRNAs during infection (Cavanagh, 1997). They are enveloped virus with a single-stranded, positive-sense RNA genome inside, which consists of a 5′ cap and a 3′ poly (A) tail (de Vries et al., 1997; King et al., 2012). They also contains the longest and the most complex RNA genome, which can be distinguished from other RNA viruses by their molecular genetics (Gorbalenya et al., 2006). So far, our knowledge about their molecular biology have mainly originated from the research progress on *Arterividae* and *Coronaviridae*.

**Figure 1:**
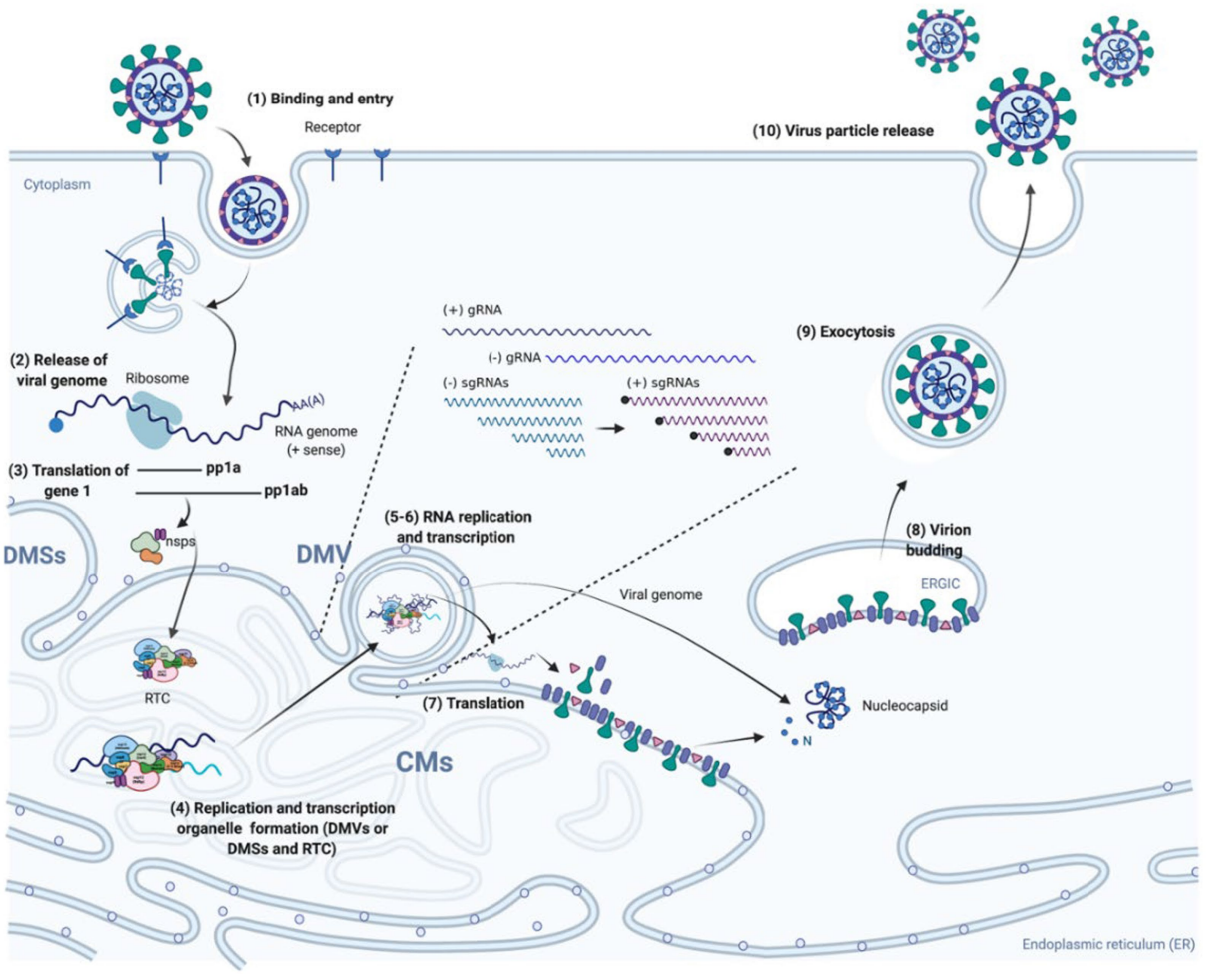
*Nidovirales* life cycle. (1) Virus particles attach to specific receptors on the surface of host cell, which enables the virus to enter into cells through direct fusion with cellular or endosomal membranes; (2–3) The incoming viral genome, a single-stranded RNA molecule, after being released and uncoated serves as template for the synthesis of two polyproteins (pp1a and pp1ab), which are afterwards cleaved by internal papain-like proteases (PLPs) and main protease (Mpro) to generate mature NSPs, including replicases; (4) The formation of viral replication/transcription organelle. The three NSPs containing TM (TM1-3) insert into the intracellular membrane to induce the formation of various membrane structures such as DMVs, CMs, and DMSs, which provide a protective microenvironment for replication and transcription. The NSPs including RdRp, primer synthetases, RNA helicase, endoribonuclease, interact with each other to form the RTC. (5–6) The gRNA and sg mRNAs are synthesized by RTC in the DMVs or DMSs and then transported outside of the DMVs or DMSs; (7) The gRNA and sg mRNAs serve as templates for synthesis of viral proteins by ribosomes; (8) Viral structural proteins are first transported into the ER membrane and then reach the ER-to-Golgi intermediate compartment (ERGIC). Once reaching at the ERGIC, the structural proteins interact with the membranes to curve it and then wrap around the N-gRNA nucleocapsid, resulting in the virion budding into secretory vesicular compartments. (9–10) Mature virus particles are transported out of the cell by exocytosis.

Nidovirus infection is initiated by the process of binding between a virus and its receptors, fusing with membranes, and releasing of the virus into the cytoplasm, in which the nucleocapsid protein is degraded to un-coat the viral genome and subsequently the uncoated gRNA is translated into polyproteins 1a and 1ab (Brian and Baric, 2005). Both polyproteins (pp1a and pp1ab) undergo auto-proteolysis by intrinsic cysteine proteases to yield 13 to 17 non-structural proteins (NSPs) (Ziebuhr et al., 2000; Fang and Snijder, 2010; Snijder et al., 2013). These NSPs are encoded by gene 1, including two proteases and three transmembrane domains (TM) containing proteins, primer synthetases, RNA-dependent RNA polymerase (RdRp), RNA helicase, and endoribonuclease. To be specific, RdRp serves as a key component in the formation of the replication-transcription complex (RTC) and plays a crucial role in the viral RNA synthesis (Hagemeijer et al., 2010; Yan et al., 2021). The RTC interacts with the modified membrane structures such as double-membrane vesicles (DMVs), tiny open double-membrane spherules (DMSs), and convoluted membranes (CMs) to carry out its functions of replication and transcription (Hagemeijer et al., 2010). The DMVs, DMSs, and CMs are derived from the endoplasmic reticulum (ER) membranes by the help of several TM containing viral NSPs (Posthuma et al., 2008; Angelini et al., 2013; van der Hoeven et al., 2016; Oudshoorn et al., 2017). They provide a protective environment for the RTC to efficiently process and synthesize RNA molecules (Roingeard et al., 2022). With the help of RTC, the negative-stranded full-length genomic RNA (-gRNA) and subgenomic RNAs (-sgRNAs) are synthesized, serving as templates to generate gRNA and sg mRNAs, which are further involved in the synthesis of replicases (pp1a and pp1ab), structural proteins, and the accessory proteins. The transmembrane structural proteins are synthesized by ER-associated ribosomes and inserted into the ER membranes, while the nucleocapsid protein (N protein) is synthesized by ribosomes in the cytoplasm. N protein binds to the viral RNA to form a stable structure called RNA-N complex, which enhances the efficiency of replication or translation (Chang et al., 2014; McBride et al., 2014). The assembly process of virion takes place in the membranes located between the ER) and Golgi apparatus (ERGIC), within which specialized compartments are formed when the membrane folds inward to create a small vesicle-like structure (Venkatagopalan et al., 2015; Boson et al., 2021). This assembly process is triggered by the interaction among the viral structural proteins and membranes (Klumperman et al., 1994; Nguyen and Hogue, 1997; de Haan et al., 1998; Wissink et al., 2005; Neuman et al., 2011; Zhang Z. et al., 2022), and the structural proteins subsequently bind to N protein and recruit the gRNA-N complex, during which the structural proteins and membranes form the outer envelope while the gRNA-N nucleocapsid is wrapped inside (Lim and Liu, 2001; Hsieh et al., 2008; Wang et al., 2009; Tseng et al., 2010; Zhang et al., 2015; Rüdiger et al., 2016; Lu et al., 2021). Once the virion assembly is completed within the ERGIC, the mature virus particles are transported out of the cell through a process called exocytosis (Figure 1).

## 2 Classification and associated diseases of *Nidovirales*

**Figure 2:**
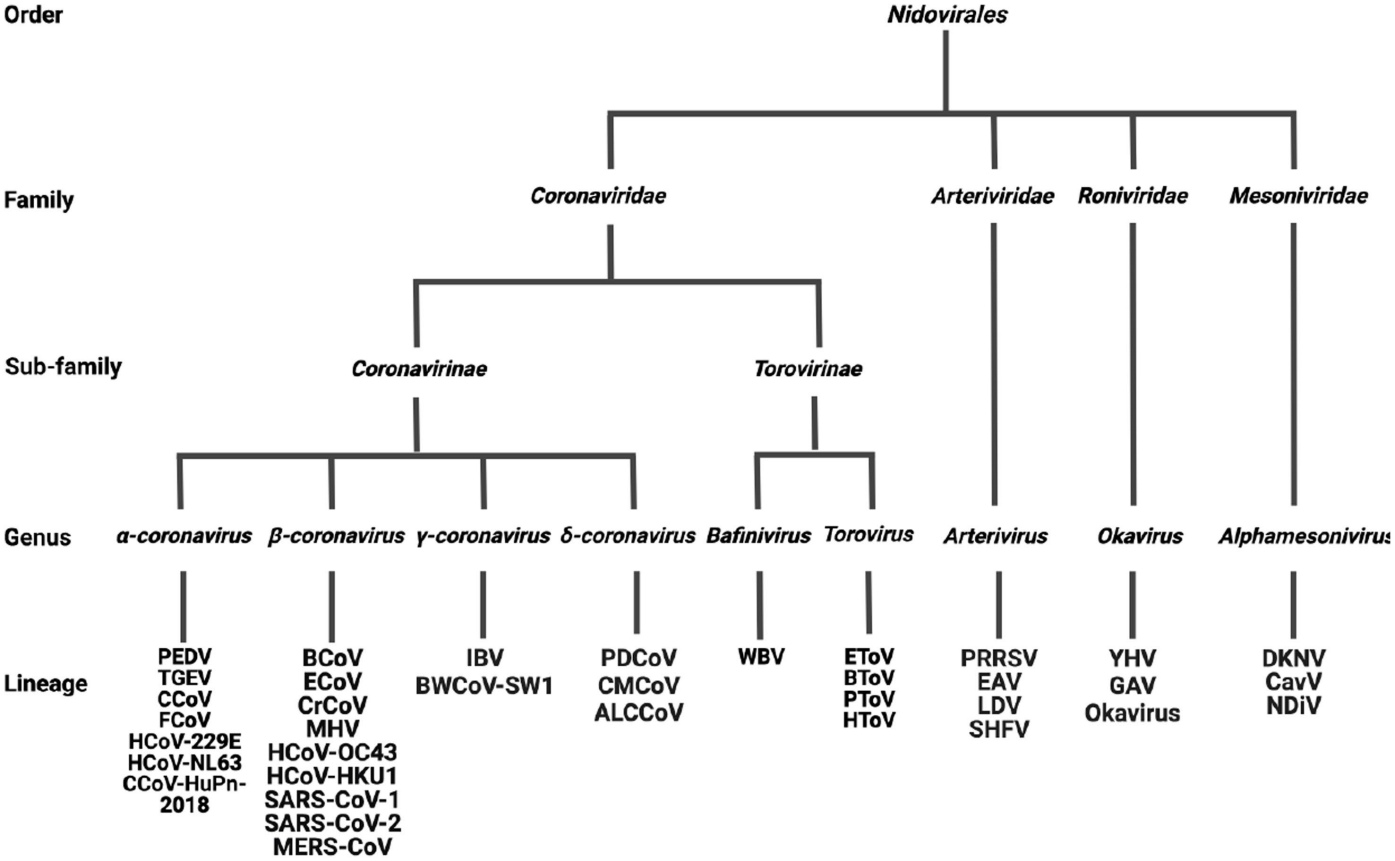
The classification of *Nidovirales*. *Nidovirales* consists of 4 families, 2 sub-families, 9 genera and a total of 36 lineages. PEDV, Porcine epidemic diarrhea virus; TGEV, Transmissible gastroenteritis coronavirus; CCoV, Canine coronavirus; FCoV, Feline coronavirus; HCoV-229E, Human coronavirus 229E; HCoV-NL63, Human coronavirus NL63; CCoV-HuPn-2018, Canine coronavirus-human pneumonia-2018; BCoV, Bovine coronavirus; ECoV, Equine coronavirus; CRCoV, Canine respiratory coronavirus; MHV, Mouse hepatitis virus; HCoV-OC43, Human coronavirus OC43; HCoV-HKU1, Human coronavirus HKU1; SARS-CoV-1, Severe acute respiratory syndrome coronavirus-1; SARS-CoV-2, Severe acute respiratory syndrome coronavirus-2; MERS-CoV, Middle east respiratory syndrome coronavirus; IBV, Infectious bronchitis virus; BWCoV-SW1, Beluga whale coronavirus SW1; PDCoV, Porcine delta-coronavirus; CMCoV, Common-moorhen coronavirus; ALCCoV, Asian leopard cat coronavirus; WBV, white bream virus; EToV, Equine torovirus; BToV, Bovine torovirus; PToV, Porcine torovirus; HToV, human torovirus; PRRSV, porcine reproductive and respiratory syndrome virus; EAV, Equine arteritis virus; LDV, Lactate dehydrogenase elevating virus; SHFV, Simian hemorrhagic fever virus; YHV, Yellow head virus; GAV, Gill-associated virus; DKNV, Dak nong virus; CavV, Cavally virus; NDiV, Nam Dinh virus.

Officially defined by the International Committee on Taxonomy of Viruses (ICTV) at the Xth International Congress of Virology (ICV) held in Jerusalem (Pringle, 1996), *Nidovirales* was classified into four families: *Arterividae, Mesoniviridae, Roniviridae,* and *Coronaviridae,* with the *Coronaviridae* further divided into two sub-families: *Coronavirinae* and *Torovirinae* (Figure 2). However, as a result of the development of virus detection technologies and viral metagenomics, an increasing number of previously unknown viruses have been discovered (Shi et al., 2018). According to the changes to virus taxonomy approved by the ICTV in 2019, currently the order *Nidovirales* is composed of eight suborders: *Abnidovirineae, Arnidovirineae, Coronadovirineae, Mesnidovirineae, Monidovirineae, Nanidovirineae, Ronidovirineae, and Tornidovirineae* (Van Regenmortel, 2000; Walker et al., 2019; Parrish et al., 2021). These eight suborders contain 14 families, 25 subfamilies, 39 genera, 65 subgenera, and 109 species. Because the newly emerged viruses have not yet been comprehensively studied yet, the following introduction on the *Nidovirales* is still based on the original taxonomy (Pringle, 1996).

*Nidovirales* can infect both vertebrates (*Coronaviridae, Arteriviridae,* and *Roniviridae*) and invertebrates (*Mesoniviridae*) (Table 1), with a wide range of host organisms, from mammal to bird, fish, crustacean, and insect (Cavanagh et al., 1994; Snijder and Meulenberg, 1998; Masters, 2006; Snijder et al., 2013; Hartenian et al., 2020; Ujike and Taguchi, 2021). Classification of viruses within the *Nidovirales* order is primarily based on several factors, including the organization of the viral genome, the homology of the genome sequence, the antigenic characteristics of the viral proteins, the replication strategy, the structure and physicochemical properties of the virus particle, the natural host range. Based on these factors, scientists and researchers are able to understand the characteristics and relationship of such viruses (Masters, 2006; King et al., 2012; Lavi et al., 2012). Compared to DNA viruses, RNA viruses lack proofreading capacity in their RdRp and thus come across more frequent errors or mutations during replication of their genetic materials, which results in their faster genetic drift and their ability to cross species barriers.

**Table 1:** The classification of *Nidovirales.*

Family	Sub-family	Genus	Virus species	Host	Genome size (kb)	Genbank accession no
*Coronaviridae*	*Coronavirinae*	*α-coronavirus*	PEDV	Swine	28.031	MK841495.1
			TGEV	Swine	28.572	DQ811788.1
			CCoV	Canine	29.051	MT114538.1
			FCoV	Feline	29.273	DQ848678.1
			HCoV-229E	Human	27.021	KU291448.1
			HCoV-NL63	Human	27.537	DQ445912.1
			CCoV-HuPn-2018	Human	29.089	MW591993.2
		*β-coronavirus*	BCoV	Bovine	31.031	U00735.2
			ECoV	Equine	30.943	OL770366.1
			CrCoV	Canine	30.876	KX432213.1
			MHV	Murine	31.291	FJ647225.1
			HCoV-OC43	Human	30.753	KU131570.1
			HCoV-HKU1	Human	29.811	MH940245.1
			SARS-CoV-1	Human	29.746	AY545919.1
			SARS-CoV-2	Human	29.903	NC_045512.2
			MERS-CoV	Human	30.031	MH734115.1
		*γ-coronavirus*	IBV	Avian	27.608	NC_001451.1
			TCoV	Avian	27.657	NC_010800.1
		*δ-coronavirus*	PDCoV	Swine	25.370	MN942260.1
			HKU11	Bulbul	26.487	NC_011547.1
			HKU12	Thrush	26.396	NC_011549.1
	*Torovirinae*	*Bafinivirus*	WBV	White bream	26.660	NC_008516.1
		*Torovirus*	EToV	Equine	a part of sequence	DQ310701.1
			BToV	Bovine	28.341	MN882587.1
			PToV	Swine	28.305	KM403390.1
			HToV	Human	a part of sequence	KJ645983.1
*Arteriviridae*		*Arterivirus*	PRRSV	Swine	15.447	AY150312.1
			EAV	Equine	12.704	MG137481.1
			LDV	Murine	14.104	NC_001639.1
			SHFV	Primate (Macaque)	15.717	NC_003092.2
*Roniviridae*		*Okavirus*	YHV	Shrimp	26.672	FJ848673.1
			GAV	Prawn	26.253	NC_010306.1
			Okavirus	Shrimp	26.662	FJ848674.1
*Mesoniviridae*		*α-mesonivirus*	DKNV	Culex	20.125	OV054251.1
			CavV	Culex	20.128	NC_015668.1
			NDiV	Vishnui	20.074	NC_020901.1

### 2.1 Coronaviridae

**Figure 3:**
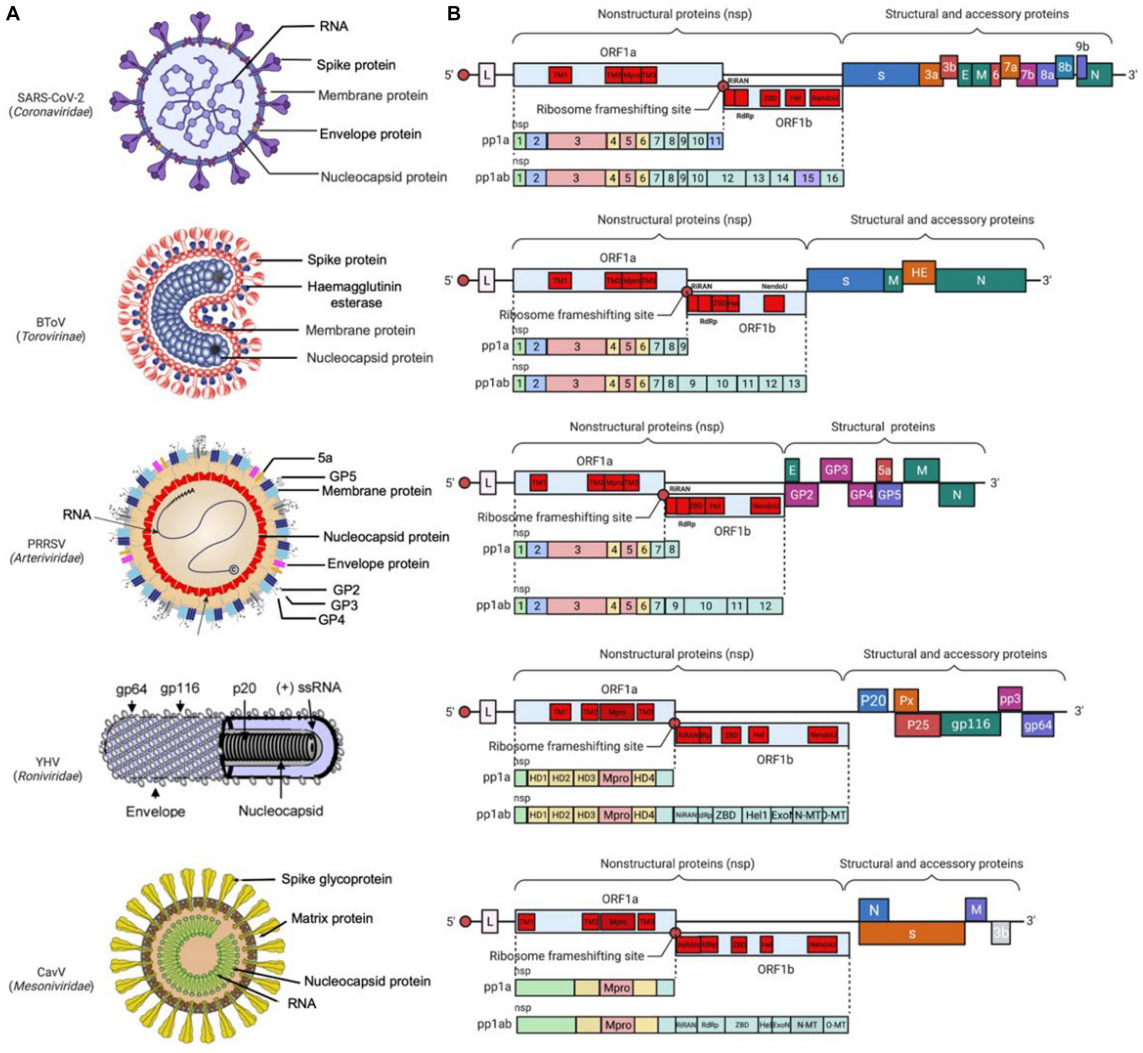
Prototype virus particle and genome structure of *Nidovirales*. **(A)** The prototype virus in each family is represented in the diagram, including SARS-CoV-2 from *Coronaviridae,* BToV from *Torovirus*, porcine reproductive and respiratory syndrome virus (PRRSV) from *Arterividae,* YHV from *Roniviridae*, and CavV from *Mesoniviridae.* **(B)** Genome organization of the prototype virus (SARS-CoV-2, BToV, PRRSV, YHV, CavV) in each family: the replicase ORF1a and ORF1b are followed by the genes encoding structural and accessory proteins. Red circles represent ribosomal frameshifting sites, and the rectangles represent the open reading frames (ORFs). Colored patterns represent domains common to all nidovirus.

*Coronaviridae* is a family of viruses with a single-stranded RNA genome in the size of 25–32 kb. The 5′-proximal two-thirds region encodes two large polyproteins (pp1a and pp1ab) to generate NSPs which are crucial for viral replication, while the 3′-proximal genome encodes 4 structural proteins which are responsible for viral entry and assembly (Brian and Baric, 2005). As well, the virus species specific accessory proteins, which play an important role in modulating the host immune response, are interspersed among the structural protein genes in the 3′-proximal region (Figure 3B).

According to the original classification, the virus family of *Coronaviridae* is further divided into two subfamilies: *Coronavirinae* and *Torovirinae* (Lai and Cavanagh, 1997). The subfamily *Coronavirinae* is named after its crown-like appearance under electron microscopy and characterized by its spherical particle structure with a diameter ranging from 80 nm to 120 nm and its surface adorned with spike (S), membrane (M), and envelope (E) protein. Inside the envelope, there is nucleocapsid which is composed of the viral genomic RNA and N protein (Figure 3A). In contrast, the subfamily *Torovirinae* is named after the Latin word “torus” which means “cushion” or “protuberance” and appears like pleomorphic, elongated particles with a characteristic “cushion-like” or “torus-like” shape, with the particle morphology including rod-shaped, kidney-shaped, and spherical particles (Ujike and Taguchi, 2021).

The subfamily *Coronavirnae* can be further divided into four genera: *α-Coronavirus*, *β-Coronavirus*, *γ-Coronavirus,* and *δ-Coronavirus.* These genera encompass a wide range of coronaviruses that infect human, mammalian animals, and avian species (Lai and Cavanagh, 1997; Masters, 2006; McCluskey et al., 2016). This subfamily of viruses mainly causes diseases characterized by symptoms in the respiratory tract, intestinal tract, liver, and central nervous system and poses severe threat not only to human health but also to livestock breeding (Drosten et al., 2003; Woo et al., 2009; Myrrha et al., 2011; Hemida et al., 2014; Zhu et al., 2020). The subfamily can be transmitted from animals to human and differ in terms of their pathogenicity, symptoms and fatality rates. Before the highly pathogenic severe acute respiratory syndrome coronavirus-1 (SARS-CoV-1), middle east respiratory syndrome coronavirus (MERS-CoV), and SARS-CoV-2 emergence, the human coronaviruses usually cause mild or moderate upper respiratory tract diseases, which include HCoV-229E (Hamre and Procknow, 1966), HCoV-OC43 (Hamre and Procknow, 1966; Vabret et al., 2003), HCoV-NL63 (van der Hoek et al., 2004), and HCoV-HKU1 (Woo et al., 2005). These viruses are generally not considered to be highly dangerous or life-threatening (El-Sahly et al., 2000; Falsey et al., 2002; Gagneur et al., 2002). Whereas, MERS-CoV, SARS-CoV-1, and SARS-CoV-2 cause severe symptoms in low respiratory tract, with the typical syndromes of fever and pneumonia (Drosten et al., 2003; Zaki et al., 2012; Salzberger et al., 2021). Among them, MERS-CoV infection is characterized by such symptoms as fever, cough, shortness of breath, and pneumonia, with a fatality rate of 34.4%; SARS-CoV infection is characterized by such symptoms as fever, chills, and body pain, and pneumonia, with a fatality rate of 9.5%; and SARS-CoV-2 infection is characterized by a wide ranges of symptoms, including fever, cough, fatigue, loss of taste or smell, pneumonia and acute respiratory distress syndrome (Petrosillo et al., 2020; Sharma et al., 2020). Since the outbreak of SARS-CoV-2 in December 2019, it has spread globally at an alarming rate, causing more than 771.5 million infections and 6.97 million deaths, with an average mortality rate of 0.9%, as reported to WHO by 25 October 2023 (Cheng et al., 2020; Petrosillo et al., 2020; Zhu et al., 2020; Salzberger et al., 2021). In fact, the real infections and deaths number are severely underestimated. As the omicron variant has become the predominant strain and the herd immunity has been established, the mortality rate of SARS-CoV-2 is continuing to decline.

Additionally, *Coronavirinae* turns out an important concern for veterinary. It is currently known that six coronaviruses infect pigs, including transmissible gastroenteritis virus (TEGV), porcine respiratory coronavirus (PRCV), swine acute diarrhea syndrome coronavirus (SADS-CoV), porcine hemagglutinating encephalomyelitis virus (PHEV), porcine epidemic diarrhea virus (PEDV), and porcine δ-coronavirus (PDCoV) (Mora-Díaz et al., 2019; Liu and Wang, 2021; Turlewicz-Podbielska and Pomorska-Mol, 2021). Specifically, TGEV，PRCV, and PHEV have been commonly found in pig herds worldwide for decades, whereas PEDV, SADS-CoV, and PDCoV have been identified more recently and cause clinically indistinguishable acute gastroenteritis, especially lethal to newborn piglets, posing significant challenges to the porcine breeding industry. As the first discovered coronavirus in the 1930’s, infectious bronchitis virus (IBV) belongs to the genus of *γ-Coronavirus* and is a highly contagious respiratory virus that causes significant economic loss in the poultry farm. It primarily infects chickens, causing respiratory signs such as depression, coughing, sneezing, nasal discharge, and even death (Najimudeen et al., 2020). Despite routine efforts in its vaccination, this virus is becoming increasingly difficult to prevent and control as a result of its high mutation rate and unpredictable emergence of diverse types throughout the world (Cavanagh, 2007; Khataby et al., 2016; Bande et al., 2017).

With a size of approximately 25–30 kb, the subfamily *Torovirinae* shares similar genome organization with *Coronavirnae* and has two genera: *Torovirus* and *Bafinivirus* (Snijder and Horzinek, 1993; Cavanagh et al., 1994; Koopmans and Horzinek, 1994; de Vries et al., 1997). Four virus species under this genus have been identified to date: equine torovirus (EToV), bovine torovirus (BToV), porcine torovirus (PToV), and human torovirus (HToV), with genetic divergence of 20–40% (Ujike and Taguchi, 2021). Infections of torovirus have been reported worldwide, with cases documented in Europe, America, Asia, New Zealand, and South Africa (Durham et al., 1989; Penrith and Gerdes, 1992; Koopmans and Horzinek, 1994; Cavanagh, 1997; Ujike and Taguchi, 2021). These viruses can cause various gastrointestinal and respiratory symptoms in their respective host species: EToV is known to cause gastrointestinal and respiratory infections in horses and is the only torovirus which has been successfully cultured *in vitro* (Kuwabara et al., 2007); BToV primarily infects cattle and cows with diarrhea and respiratory symptoms (Ito et al., 2009; Aita et al., 2012; Lojkic et al., 2015); PToV mainly brings about gastrointestinal infection in pigs，with its coinfections with other pathogens usually exacerbating the symptoms (Hu et al., 2019); HToV associates with gastroenteritis and diarrhea in children as well as necrotizing enterocolitis in infants (Durham et al., 1989). The genus of *Bafinivirus* that infects fish was discovered in 2006 (Schütze et al., 2006).

### 2.2 Arteriviridae

The *Arteriviridae* was established as a distinct family in 1996; later on, it was grouped into the order *Nidovirales* because it shares similarities with the family *Coronaviridae* (Cavanagh, 1997; Snijder and Meulenberg, 1998; Gorbalenya et al., 2006; King et al., 2012). Arterivirus particles are spherical and enveloped, with a core housing the RNA genome of approximately 12.7–15.7 kb (Plagemann and Moennig, 1992). Unlike coronaviruses, arteriviruses do not have an obvious spike protein on their surface. Instead, they have relatively small protrusions. The envelope consists of several proteins, including two major envelope proteins GP5-M heterodimer, three minor proteins GP2-GP3-GP4 heterotrimer and GP2-GP4 heterodimer, and two minor protein E and 5a (Snijder et al., 2013). All envelope proteins are critical for producing infectious progeny (Dea et al., 2000; Wissink et al., 2005; Music and Gagnon, 2010). Inside the envelope, there is RNA genome wrapped with N protein, which form a pleomorphic core with a mean diameter of 39 nm (Spilman et al., 2009; Dokland, 2010) (Figure 3A). The diame1ter of the virus particles, observed under the cryo-electron microscopy, was approximately 50–60 nm, significantly smaller than coronaviruses (Spilman et al., 2009).

The *Arteriviridae* primarily infect mammals, including equid, swine, opossum, non-human primate, and rodent (Plagemann and Moennig, 1992). One notable member of this family is the prototype equine arteritis virus (EAV), which was firstly discovered in 1957 and is known to infect horses (Bryans et al., 1957; Del Piero, 2000; Balasuriya et al., 2013). Another two species of lactate dehydrogenase-elevating virus (LDV) and simian hemorrhagic fever virus (SHFV), were firstly isolated more than 50 years ago. SHFV is known to cause a highly lethal fever in African non-human primates while LDV infects mice (Riley et al., 1960; Notkins and Shochat, 1963; Plagemann et al., 1995; Snijder and Meulenberg, 1998; Brinton et al., 2015). The porcine reproductive and respiratory syndrome virus (PRRSV) is a highly contagious virus that infect pigs, and its emergence causes significant economic losses to the global swine industry (Neumann et al., 2005; Tian et al., 2007; Lunney et al., 2016; Guo et al., 2018; Zhang H. et al., 2022). Arteriviruses are transmitted through respiratory routes or body fluids; in most cases, they affect macrophages. They can cause a range of symptoms, including persistent or acute asymptomatic infections, miscarriage, respiratory disease, arthritis, fatal hemorrhagic fever, and polio (Snijder et al., 2013). For example, EAV and PRRSV are known to cause mild-to-severe respiratory disease and lead to abortion in pregnant animals (Balasuriya and Carossino, 2017). Due to their veterinary importance, EAV and PRRSV have been characterized extensively, severing as the basis for our current understanding of arterivirus.

### 2.3 Roniviridae

The family *Roniviridae* include a single genus called *Okavirus* which infect crustacean, mostly shrimp (Cowley et al., 2000; Walker et al., 2021). The virus particles are enveloped with bacilliform geometries and helical symmetry, with a diameter of around 40–60 nm and a genome of 26–27 kb inside (Dhar et al., 2004; Figure 3A). The genus *Okavirus* includes gill-associated virus (GAV) and yellow head virus (YHV), which have been found to associate with mortality in cultured black tiger prawns (*Penaeus monodon*) and white Pacific prawns (*Penaeus vannamei*). It has been reported that these viruses caused economic loss in shrimp farm in Eastern Australia, Thailand, and China (Cowley et al., 2001; Munro and Owens, 2007; Munro et al., 2011; Dong et al., 2017). They have been listed as notifiable pathogens by the World Organization for Animal Health.

### 2.4 Mesoniviridae

Found in 2011, the *Mesoniviridae* has only one genus called *α-Mesonivirus*, which are mosquito-specific virus with a wide geographic distribution (Nga et al., 2011; Zirkel et al., 2011; Lauber et al., 2012; Vasilakis et al., 2014). Belonging to the genus *α-Mesonivirus*, Cavally virus (CavV) and Nam Dinh virus (NDiV) have been the first two characterized mesoniviruses (Nga et al., 2011; Zirkel et al., 2011). These two viruses are closely related and belong to the same species, *α-Mesonivirus* 1. Other phylogenetically diverse mesoniviruses are isolated from a range of mosquito species and geographic locations (Kuwata et al., 2013; Thuy et al., 2013; Zirkel et al., 2013). The mesonivirus particles are about 120 nm in diameter, with rod-shaped spike protein (77 kDa) and differentially glycosylated membrane proteins (17, 18, 19, 20 kDa) on the surface, and a nucleocapsid (25 kDa-N protein and 20 kb-RNA genome) inside (Figure 3A).

## 3 RNA genome

The *Nidovirales* genome have been studied extensively in recent decades (de Vries et al., 1997; Gorbalenya et al., 2006). Despite variations in genome size and virus particle morphology, *Nidovirales* have been classified primarily based on their common genetic organization, a set of conserved domains and enzyme functions within the polyproteins, and their unique transcription strategy (King et al., 2012). The 5′-proximal genome contains two ORFs (ORF1a and ORF1b), which encompass two-thirds of the genome and encode two giant polyproteins pp1a and pp1ab. These two ORFs possess a conserved domain backbone, including their domains in the sequential order as follows: 5′-TM1-TM2-Mpro-TM3-NiRAN-RdRp-ZBD-HEL-NendoU-3′ (the first four in ORF1a while the remaining in ORF1b) (King et al., 2012). The 3′-proximal genome contains smaller ORFs, which encode structural proteins and accessory proteins, as translated from co-terminal sg mRNAs. These ORFs are different in number, size, and length in different families, and some accessory genes are unique to certain virus species, especially in coronaviruses (de Vries et al., 1997; Figure 3B). In addition, at the 5′- and 3′-termini, there are untranslated regions (UTR) which can regulate the viral RNA replication and transcription.

After virus entry and uncoating, the gRNA is released into the cytosol, and directly serves as a template for transcription, enabling cap-dependent translation of ORF1a to produce pp1a. In addition, an RNA pseudoknot structure is located near the end of ORF1a together with a slippery sequence of “UUUAAC,” enabling −1 or −2 ribosomal frameshift and translation on ORF1b to produce a longer pp1ab (Brierley et al., 1989; Plant et al., 2005; Patel et al., 2020). Pp1a and pp1ab are cleaved by intrinsic proteases, either co-translationally or post-translationally, to generate mature NSPs, including papain-like proteases (PLPs), NSPs with TM (TM1, TM2, TM3), 3C-like main protease (Mpro), nucleotidyltransferase (NiRAN), RdRp, superfamily 1 helicase (ZBD-HEL), and endoribonuclease (NendoU) (Gorbalenya et al., 1989; Ziebuhr et al., 2000; Li et al., 2015). The proteins encoded by ORF1a play a significant role in modulation of host gene expression, cleavage and maturation of pp1a and pp1ab (PLP and Mpro) (Ziebuhr et al., 2000), and modification of host membranes so as to create an environment suitable for viral genome synthesis (NSPs with TM1, TM2, or TM3) (van der Hoeven et al., 2016), while the proteins encoded by ORF1b play an important role in RNA replication and transcription, including NiRAN, RdRp, HEL, and NendoU, referred to as replicases (Liu et al., 1994; Thiel et al., 2001; Fang and Snijder, 2010; Lehmann et al., 2015c; Hu et al., 2021). Next, we focus on reviewing these replicases.

## 4 Replicases

### 4.1 PLP and 3CLpro (Mpro)

Nidoviruses encode multiple PLPs and a 3CLpro (also called Mpro) (Ziebuhr et al., 2000; Snijder et al., 2013). The PLPs are located in the upstream of TM1 and are present in NSP2 for arteriviruses and in NSP3 for coronaviruses (Snijder et al., 1995; Kanjanahaluethai and Baker, 2000; Ziebuhr et al., 2001; Harcourt et al., 2004). The number of active PLP domains can vary from one virus species to another (Vatter et al., 2014). Structural and enzymatic studies reveal that the PLPs not only cleave peptide bonds (NSP1/NSP2/NSP3 junctions in arteriviruses and NSP1/NSP2/NSP3/NSP4 junctions in coronaviruses) but also act as a deubiquitinating (DUB) enzyme to remove Lys63-linked ubiquitin chains or ubiquitin-like modifiers (ISG15) from host substrates, antagonizing the innate immune response (Frias-Staheli et al., 2007; van Kasteren et al., 2012). Mpro, a chymotrypsin-like protease that plays the main role in the polyprotein processing, is located in NSP4 for arteriviruses and NSP5 for coronaviruses, respectively (Tian et al., 2009). Mpro cleaves all sites downstream of NSP3 for arteriviruses and downstream of NSP4 for coronaviruses (Ziebuhr et al., 2000; Tian et al., 2009). As these proteases facilitate the cleavage of pp1a/pp1ab and maturation of the replicases, the conserved functional enzymatic structures are becoming the optimal therapeutic targets (Dai et al., 2020; Khare et al., 2020; Zhang L. et al., 2020; Banerjee et al., 2021; Capasso et al., 2021). For example, the FDA authorized Paxlovid (nirmatrelvir/ritonavir) specifically targets at the Mpro of SARS-CoV-2 to block the enzymatic function, thereby effectively reducing virus replication and resulting in a great decrease of hospitalization and death among the COVID-19 patients (Graham, 2021; Cokley et al., 2022; Marzolini et al., 2022; Amani and Amani, 2023; Harris, 2023).

### 4.2 Transmembrane proteins

ORF1a encodes three TM proteins (TM1, TM2, and TM3), namely NSP2, NSP3, NSP5 in arteriviruses and NSP3, NSP4, NSP6 in coronaviruses. TM1 and TM2 reside upstream of the Mpro, while TM3 is located at its downstream (Figure 3B). All of the TM proteins span across the membrane more than once. These TM proteins play a crucial role in modifying intracellular membranes to form DMV, DMS, or CM, which accommodate the RTC and associate with viral RNA synthesis (Bost et al., 2000; Snijder et al., 2006; Posthuma et al., 2008; Angelini et al., 2013; Hagemeijer et al., 2014; van der Hoeven et al., 2016; Oudshoorn et al., 2017). However, their biological significance needs to be further studied.

### 4.3 RNA-dependent RNA polymerase (RdRp)

**Figure 4:**
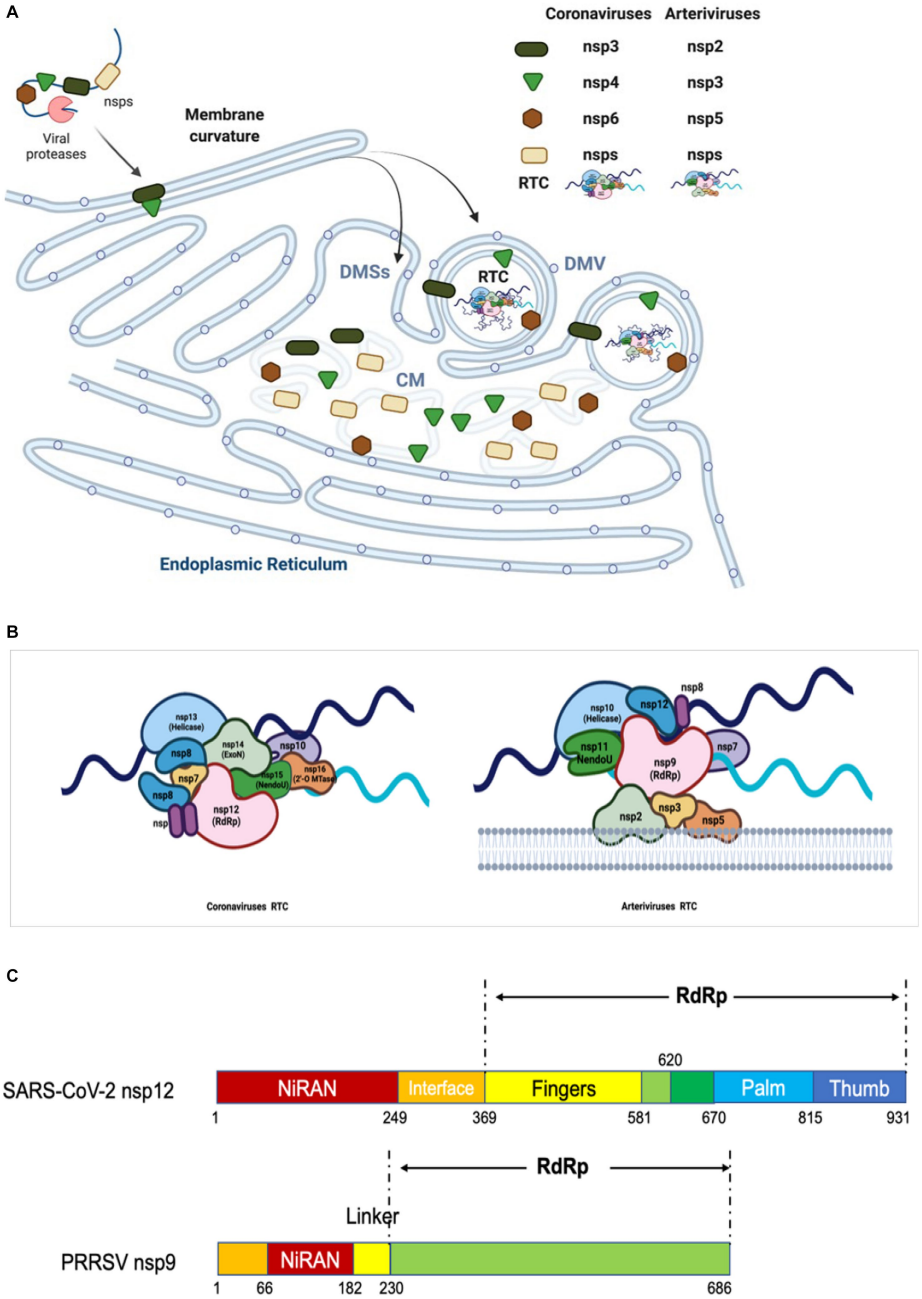
Diagram of replication/transcription organelle and RTC. **(A)** Diagram of CMs/DMVs/DMSs. Nidovirus infection leads to the rearrangement of ER membranes and the envelopment of RTC. During this process, the NSPs containing TM are cleaved from pp1a and pp1ab, and then embedded in the ER membrane to form a membrane fold and luminal loops. These interactions yield a complex array of CMs, DMSs or DMVs, which are contiguous with ER membranes. The replicases cleaved from pp1a and pp1ab interact each other and form RTC in DMV. **(B)** Model of RTC. The core RTC is composed of the RdRp (coronavirus: NSP12; arterivirus: NSP9), processivity factors (coronavirus: NSP7-9; arterivirus: NSP12), ExoN complex (coronavirus: NSP10 and NSP14), NendoU (coronavirus: NSP15; arterivirus: NSP11), and helicase (coronavirus: NSP13; arterivirus: NSP10). As shown, for coronavirus, the NendoU is the center of the RTC complex, capped on the two sides by NSP14/NSP16/(NSP10)_2_, which afterwards recruits NSP12/NSP7/(NSP8)_2_ to the complex. Helicase is around the ExoN complex. The model is based on the known structure and interactions between the proteins (243). **(C)** The functional domains of RdRp of coronavirus and arterivirus are shown.

RdRp is encoded by the N-terminal region of ORF1b (the enzymatic domain is present in NSP9 for arteriviruses and NSP12 for coronaviruses). It is the critical component of RTC to catalyze the synthesis of RNA with the help of helicase, NendoU, and ribose-2’-O-methyltransferase (O-MTase), and thus plays a crucial role in the replication and transcription (Subissi et al., 2014; Lehmann et al., 2016). Our current knowledge of the structural and enzymatic characteristics of RdRp primarily comes from studies conducted on SARS-CoV and SARS-CoV-2 (Kirchdoerfer and Ward, 2019; Gao et al., 2020). RdRp consists of three domains: a canonical RdRp core domain at C-terminus (occupying two-thirds of the protein), a NiRAN domain at N-terminus, and an interface domain (Figure 4C) (Lehmann et al., 2015a; Posthuma et al., 2017).

The RdRp core domain can be further divided into three subdomains: fingers, palm, and thumb subdomains. In the active site there are multiple functional motifs (6 in arteriviruses and 7 in coronaviruses), named A-G respectively, which are responsible for recognizing the RNA template and substrate, as well as catalyzing the condensation of nucleotides (Poch et al., 1989; Lehmann et al., 2016; Kirchdoerfer and Ward, 2019; Gao et al., 2020). The motif A contains the divalent-cation-binding residues D (D619 and D623 in SARS-CoV-1 NSP12, D618 in and D623 in SARS-CoV-2 NSP12, and D445 and D450 in EAV NSP9), while motif C contains the conserved catalytic residues SDD sequence (residues 759–761 in SARS-CoV-1 and SARS-CoV-2 NSP12, and residues 559–561 in EAV NSP9). The RNA template is supposed to enter the active site in motifs A and C, and the NTP entry channel is within motif F, as determined by the cryo-electron microscopy structure of SARS-CoV-2 NSP12 (Gao et al., 2020). With its key role in viral RNA replication and transcription, RdRp is considered as a primary target for antiviral inhibitors that are designed to mimic nucleotides and inhibit viral RNA replication and transcription. For example, remdesivir is nucleotide analogs which can interfere with the action of RdRp and arrest the RNA synthesis process by delaying the elongation (Gordon et al., 2020a,b). It is the first drugs authorized by FDA for treatment of COVID-19.

As a unique and essential enzymatic domain of *Nidovirales* (Lehmann et al., 2015a), the N-terminal NiRAN domain possesses a self-nucleotidylating activity, which is important for viral RNA replication. It is speculated that the NiRAN might perform multiple functions, for example, it may act as an RNA ligase in mRNA capping (Yan et al., 2021), or serve in protein-primed initiation of RNA synthesis by transferring a NMP to another viral protein NSP9 (Wang B. et al., 2021). These hypotheses have been extensively discussed in a study by Lehmann et al. (2015a).

### 4.4 Helicase

Based on structural and functional characteristics, helicases can be classified into two subfamilies-SF1 (ZmHEL1) and SF2. The 5′-to-3′ helicase (NSP10 for arteriviruses and NSP13 for coronaviruses) belong to SF1 (ZmHEL1). This helicase domain is exceptionally located downstream of the RdRp in *Nidovirales* (Gorbalenya et al., 1989), while in other families of positive sense single-stranded RNA viruses, it is located upstream of RdRp. As a helicase, ZmHEL1 possesses a conserved N-terminal zinc-binding domain that is commonly found in nidoviruses and is involved in unwinding activity (Seybert et al., 2005; Shi et al., 2020; Tang et al., 2020; Maio et al., 2023). The helicase activity combined with NTP-binding activity plays the vital role in unwinding RNA molecules in the 5′-3′ direction (Seybert et al., 2000a; Ivanov et al., 2004b; Shu et al., 2020; Ren et al., 2021; Fang et al., 2023).

### 4.5 Endoribonuclease (NendoU)

NendoU is a protein that is specific to *Nidovirales*, including NSP11 for arteriviruses and NSP15 for coronaviruses, a conserved protein that does not have counterparts in other RNA viruses, thus serving as a diagnostic molecular marker for nidovirus (Ivanov et al., 2004a; Gorbalenya et al., 2006; Deng and Baker, 2018). One of its functions of is to cleave the poly (U) sequence which are produced during genome replication/transcription and are found in viral −RNA intermediates, thereby regulating the ratio of −RNA to +RNA and reducing the accumulation of dsRNA. In this way, this protein facilitates the genome replication and synthesis of sg mRNA and also contributes to the evasion of IFN response by reducing the accumulation of dsRNA accumulation (Hackbart et al., 2020; Gao et al., 2021).

### 4.6 Other replicases

In addition to above conserved replicases, several other conserved replicases that perform RNA synthesis and processing have been identified in some but not all nidoviruses (Posthuma et al., 2017). There are two clades of nidoviruses: one with a large genome size ranging from 25 to 31.7 kb (*Coronaviridae*, *Torovirinae*, and *Roniviridae*), and the other with a small genome size ranging from 12.7 to 15.7 kb (*Arterividae*) (Gorbalenya et al., 2006). As a result, the replicase polyproteins of large nidoviruses contain two specific activities that are not found in *Arterividae*, namely 3′-5′exoribonuclease (ExoN) encoded by NSP14 (Chen Y. et al., 2009; Tahir, 2021) and O-MTase encoded by NSP16 (Decroly et al., 2011). ExoN is essential for the high fidelity of long RNA synthesis, while O-MTase helps to add the cap structure at the 5′-terminus of viral RNA (Posthuma et al., 2017). The mimicking of host mRNA cap helps the virus to escape the host detection, thereby evading the innate immune responses (Chen and Guo, 2016). The importance of NSP16 for coronavirus infection and pathogenesis makes it becoming an attractive target for antiviral therapeutic treatment (Menachery et al., 2014; Chang and Chen, 2021; Tahir, 2021).

The other two RNA-processing domains conserved in *Coronavidae* and *Torovirinae* are ADP-ribose-1′-phosphatase (ADRP) and nucleotide cyclic phosphodiesterase (CPD) respectively (Snijder et al., 2003; Draker et al., 2006). The ADRP domain encoded by ORF1a plays a role in the cellular RNA processing pathway by removing the phosphate from the adenosine diphosphate ribose 1′-phosphate substrate (Michalska et al., 2020). However, the highly specific phosphatase activity is not essential for viral replication, which has been demonstrated by substitutions of active-site residues or complete deletion of the ADRP domain (Putics et al., 2005; Hurst-Hess et al., 2015). The ADRP domain is found in the similar position NSP3 of both families *Coronavidae* and *Torovirinae*, indicating that this region is inherited from a common ancestor. Whereas, the CPD domain is only present in the family *Torovirinae* and *Coronavidae* MHV. In MHV, the CPD domain is expressed by ORF2 though deletion of the CPD-encoding ORF2 does not affect the replication of MHV *in vivo* (Schwarz et al., 1990); however, the other study shows that the mutation of MHV ORF2 causes a attenuated form of the virus in its natural host (Sperry et al., 2005). Thus, the CPD domain of MHV may be one of the determinants of virus pathogenicity. In *Torovirinae*, this enzyme is encoded by the 3′ end of replicase of ORF1a and located immediately upstream of the ORF1a/ORF1b junction, which is involved in the processing of viral RNA (Snijder et al., 1991).

## 5 RNA synthesis

### 5.1 Formation of DMVs

*Nidovirales* replicates its genome in the ER membrane-associated structures known as “replication organelles,” which include a complex vesculo-tubular network of CMs, DMVs, and DMSs, partly interconnected through their outer membranes. The SARS-CoV dsRNA is mainly found inside the DMVs (Knoops et al., 2008), while the newly synthesized MHV RNA is located in close proximity to both DMVs and CMs (Gosert et al., 2002); furthermore, the viral RNA positively correlates with the number of DMVs (Ulasli et al., 2010). Above observations suggest that both DMVs and CMs serve as sites for the synthesis of viral RNA.

The rearrangement of the host membranes is important to create a micro-environment appropriate for the synthesis of viral RNA and the recruitment of host factors. The association of viral RNA synthesis with membrane structures exhibits several advantages: (1) An appropriate environment is created by anchoring the viral replicases necessary for replication and transcription; (2) By anchoring viral replication complex to membrane structures, the environment is created for the diffusion of metabolites and macromolecules, so as to facilitate the synthesis process; (3) Compartmentalization helps to separate and organize the process of replication/transcription, translation, and packaging, which ensures that these processes occur in a coordinated manner. It also creates a protected environment for viral RNA replication by eluding recognition and degradation of RNA in the cytosol; (4) The insulation of the RNA replication/transcription intermediates, such as dsRNA, could hinder or delay the host’s innate immune response (Neufeldt et al., 2016; van der Hoeven et al., 2016).

The DMVs (about 100 nm) of arterivirus was firstly observed in the perinuclear region of the cell with the electron microscopy since 1970s (Wood et al., 1970), as so observed in other members of this family later on (Stueckemann et al., 1982; Wada et al., 1995; Weiland et al., 1995; Pol et al., 1997; Pedersen et al., 1999). These DMVs are connected to reticular regions of CMs between them, and contiguous with the membrane donor ER. Additionally, the ribosomes are close to the outer membrane of the DMVs (Wood et al., 1970; Stueckemann et al., 1982; van der Hoeven et al., 2016).

The expression of EAV NSP2 and NSP3 (contain TM1 and TM2) is sufficient to induce the DMVs formation and NSP3 plays a key role in the remodeling of membranes (Posthuma et al., 2008); however, the presence of additional NSP5 results in the production of DMVs whose size is more homogenous and closer to those formed in EAV-infected cells (83 + −21 nm, *n* = 145) (Figure 4A), indicating a regulatory role for NSP5 in regulating the membranes curvatures and formation of DMVs (van der Hoeven et al., 2016). The NSP2, NSP3 and NSP5 of arterivirus are believed to serve as DMVs scaffolding proteins to recruit other components of RTC to the replicase site (Pedersen et al., 1999; Posthuma et al., 2008; van der Hoeven et al., 2016).

Diverse coronaviruses induce similar membrane structures, including DMVs and DMSs (Snijder et al., 2020; Zhang J. et al., 2020). The DMVs are around twice in the diameter and 8-fold in the volume of those arterivirus-induced DMVs. The SARS-CoV induced DMVs connect with other DMVs and also connect to the ER through their outer membranes (Knoops et al., 2008). 3D electron microscopy reconstructions and living cell imaging also show that SARS-CoV-2 induced DMVs are tethered to the ER, with alteration of the mitochondrial network, remodeling of cytoskeleton elements, and recruitment of peroxisomes to DMVs (Cortese et al., 2020). The *γ-Coronavirus* IBV-induced DMVs are either tethered to the zippered ER with channel connecting the interior of the DMVs with the cytoplasm, or exists as isolated vesicles without DMV-DMV or DMV-ER connections (Maier et al., 2013; Doyle et al., 2018). The use of H3-uridine to metabolically label the newly synthesized molecules enables researcher to reaffirm that DMVs provide an optimal environment for virus RNA synthesis (Snijder et al., 2020), which is further supported by the use of specific antibodies to bind to the target molecules, such as dsRNA and DMVs (Knoops et al., 2008). Recent findings from cryotomography reveal the presence of membrane-spanning hexameric, crown-shaped pore complex in MHV induced DMVs, which makes the viral RNA exporting from DMVs possible. The observation of nucleocapsid structure on the cytosolic side of the DMVs demonstrates that the RNA is exported from DMVs for encapsidation (Wolff et al., 2020).

For coronavirus, the formation of DMVs can be induced by co-expression of NSP3, NSP4, and NSP6 (Angelini et al., 2013; Oudshoorn et al., 2017), which contain three conserved TMs respectively, and are functionally analogous to arterivirus NSP2, NSP3, and NSP5 (Gorbalenya et al., 2006; Figure 4A). The co-expression of three SARS-CoV NSPs (namely NSP3, NSP4, and NSP6) forms both DMVs and other structures resembling the CMs and DMSs presented in SARS-CoV infection (Angelini et al., 2013). Report also shows that MERS-CoV NSP3 and NSP4 can rearrange the cellular membranes to generate DMVs where the RTC is assembled and anchored (Oudshoorn et al., 2017). Co-expression of SARS-CoV-2 NSP3 and NSP4 also generate DMVs, whereas NSP6 zippers ER membrane and forms the connectors. NSP6 may act as filter in communication between the DMVs and the ER, organizer of DMV cluster, or may mediate contact with lipid droplets (Ricciardi et al., 2022). The co-expression of IBV NSP3, NSP4, NSP6 generates DMVs, and NSP4 alone is sufficient to induce membrane pairing, but not fully resembles DMVs (Doyle et al., 2018).

In both arteriviruses and coronaviruses, it is likely that the proteins containing TM1 and TM2 can induce the formation of DMVs, while the protein comprising TM3 may only modulate the formation of DMVs. Recently, it has been found that ER proteins VMP1 and TMEM41B contribute to DMV formation by facilitating NSP3-NSP4 interaction and ER zipping or subsequent closing of DMVs; the phosphatidylserine (PA) levels is also important for DMV formation (Ji et al., 2023). More and more evidences show that autophagy machinery and ER-associated degradation machinery are hijacked by coronavirus for the DMV formation (Prentice et al., 2004; Twu et al., 2021; Liang et al., 2022; Tan et al., 2023). It should be further investigated whether more host factors are involved in the formation of DMV.

### 5.2 Formation of RTC

For efficient replication, multiple replicases interact each other to form RTC and then the RTC attaches to modified intracellular membranes, resulting in the formation of a membrane-bound complex which is responsible for RNA synthesis (Sawicki et al., 2005). The association of RTC with modified intracellular membranes is a feature commonly observed in the positive-stranded RNA viruses that infect animals. As for coronavirus, a set of replicases (NSP7, NSP8, NSP9, NSP10, NSP12, NSP13, NSP14, NSP15, and NSP16) assemble into the RTC which is responsible for synthesizing the negative-stranded intermediates, gRNA, and sg mRNAs (Hagemeijer et al., 2010; Subissi et al., 2012; Kirchdoerfer and Ward, 2019; Chen et al., 2020; Hillen et al., 2020; Peng et al., 2020; Wang et al., 2020; Yan et al., 2020, 2021; Mishchenko and Ivanisenko, 2022). The NSP7-NSP8-NSP12 complex plays a central role in the replication/transcription process of coronavirus. NSP12 contains the RdRp domain in its C-terminal region, and serves as the key enzyme for catalyzing the incorporation of NTPs into the growing RNA chain. In collaboration with NSP7 and NSP8, NSP12 forms a holoenzyme RdRp (holo-RdRp) to drive the RNA synthesis in primer-dependent manner (te Velthuis et al., 2010; Ahn et al., 2012; Kirchdoerfer and Ward, 2019; Peng et al., 2020). The primers for the RNA replication/transcription are synthesized by NSP8, which bears a noncanonical RdRp activity and acts as an RNA primase (Imbert et al., 2006; te Velthuis et al., 2012; Biswal et al., 2021). Other subunits have supporting roles in the RTC. When this complex is working, two subunits of NSP13 are positioned above the RTC in which one subunit binds to the 5′ end of the RNA template downstream at the NSP12 RdRp active site for 5′-3′ nucleic acid unwinding (Perry et al., 2021). As a unique ExoN encoded by coronavirus, NSP14 forms an RNA proof reading complex together with NSP10 (Denison et al., 2011; Tahir, 2021). The mismatched base is directed into the shallow active site of the ExoN domain in which it interacts with conserved catalytic residues. Meanwhile, a portion of the dsRNA molecule interacts with both the N-terminus of NSP10 and the residues which are located outside the catalytic site of NSP14-ExoN (Ferron et al., 2018). Associated with RTC, the NSP15 is responsible for cleaving the −RNA intermediates to adjust the ratio of +RNA to −RNA, and reduce the level of dsRNA so as to help the virus escape the host innate immune response (Athmer et al., 2017; Deng and Baker, 2018; Gao et al., 2021; Perry et al., 2021). NSP16 is located in the RTC and responsible for the RNA capping together with NSP10 (Snijder et al., 2016; Benoni et al., 2021). NSP9 inhibits and controls the catalytic activity by inserting into the catalytic center of NSP12 (Slanina et al., 2021; Yan et al., 2021) (Figure 4B).

Similar to coronavirus, the assembly of RTC in arterivirus also requires multiple NSPs to work together. By examining location of PRRSV NSPs during infection, Song et al. found that NSP2, NSP4, NSP7, NSP8, NSP9, NSP10, NSP11, and NSP12 were colocalized well with dsRNA which reveals the virus replication sites, indicating all these NSPs are located to viral RTC (Song et al., 2018). Although NSP3, NSP5, and NSP6 were not examined due to lack of antibodies, the interaction among NSP2, NSP3 and NSP5 suggests that NSP3 and NSP5 are associated with RTC. The core components of arterivirus RTC are possibly composed of all NSPs encoded by ORF1b, including NSP9, NSP10, NSP11, and NSP12 (Snijder et al., 2013). The NSP2, NSP3, NSP5 are responsible for the formation of DMVs and interact with other NSPs to recruit the RTC core components (Nan et al., 2018). The C-terminus of NSP9 involves the function of RdRp, while the N-terminal of NSP9 has been discovered to contain a domain called RdRp-associated NiRAN (Lehmann et al., 2015a, 2016) (Figure 4C). NSP10 is the RNA helicase which can unwind the secondary structure of RNA (Seybert et al., 2000b; Bautista et al., 2002; Lehmann et al., 2015c). Sharing the same/with their own homologs across diverse families of nidoviruses, both NSP9 and NSP10 serve as the key virulence determinants of PRRSV (Li et al., 2014). NSP11 is a protein belonging to the NendoU family and its catalytic sites are highly conserved in *Nidovirales*, although its function remains poorly defined in the arterivirus life cycle (Nedialkova et al., 2009; Zhang et al., 2017). NSP12, a protein that is specific to arterivirus and plays an unexpected key interaction role, can interact with NSP1β, NSP2, NSP9, NSP10, and NSP11, and colocalize well with the DMVs (Lehmann et al., 2015b; Song et al., 2018). During infection, the NSP7 and NSP8 associate with the RTC, and the NSP7 interacts with NSP9; however, their functions in virus replication are poorly understood (Li et al., 2012; Chen et al., 2017). NSP6 has been shown to interact with NSP12; however, due to the unavailability of antibodies and the small size (16 aa), whether NSP6 is involved in RTC has not been determined yet (Kappes and Faaberg, 2015; Song et al., 2018). In all, NSP2, NSP3, and NSP5 form the scaffold of DMVs for supporting the RTC core components in binding to the DMVs, while NSP9 and NSP12 combine together to form a central hub (like a substrate pocket) which is connected to other replicases, including NSP7, NSP8, NSP10, NSP11(Figures 4A,B) (Song et al., 2018).

### 5.3 Synthesis of RNA

The RNA-dependent RNA synthesis takes place within the cytoplasm of the infected cells and is facilitated by a complex called RTC that consists of viral replicases and host factors (Lai and Cavanagh, 1997; Enjuanes et al., 2006). During the synthesis process, the viral genome is replicated to produce a full-length gRNA which can play multiple functions: acting as a template for translation of viral replicases (pp1a and pp1ab); serving as a template for synthesis of −RNA intermediates; and working as the genome that will be packaged into new viral particles. Additionally, RTC is also involved in the transcription to yield a nested set of sg mRNAs which are responsible for expressing the viral structural and accessory proteins.

#### 5.3.1 Replication of viral genome

Similar to other +RNA viruses, the replication of nidovirus genome is a continuous process mediated by synthesizing a full-length −RNA (Sola et al., 2015). Firstly, the viral genome acts as a template to translate the viral replicases (pp1a and pp1ab) which are further processed into more than 10 NSPs by internal enzymatic cleavage. The newly synthesized hydrophobic NSPs with TM trigger the formation of DMVs or DMSs, and further recruit other NSPs to form functional RTC. Secondly, the synthesis of −RNA intermetiates begins at the 3′-terminus of the viral genome and is facilitated by the RTC, under the help of the 3′-terminal RNA sequence and the secondary RNA structures. Thirdly, under the help of the catalytic RTC enzyme, the full-length complementary −RNA is synthesized and in turn serves as the template for producing gRNA (Snijder et al., 2016).

#### 5.3.2 Transcription of sg mRNAs

**Figure 5:**
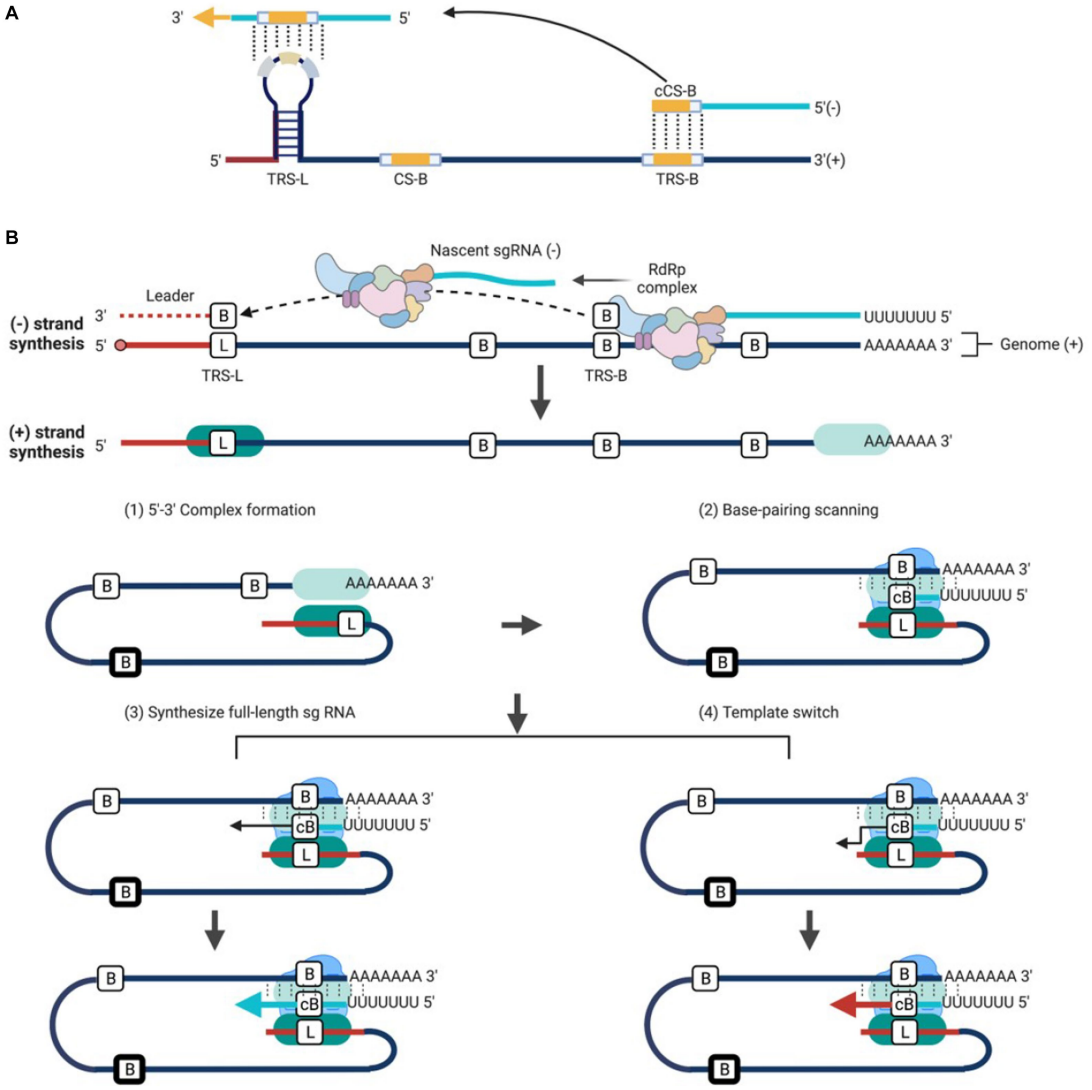
Working model of discontinuous transcription for *Nidovirales*. **(A)** TRS contains a conserved 6–7 nt core sequence (CS) flanked by 5′ and 3′ flanking sequences. TRS-B precedes each body gene, containing the core sequence (CS-B) and variable 5′ and 3′ flanking sequence. TRS-L is present at the 5′ end of the genome in an exposed location, with the leader core sequence (CS-L). **(B)** Three-step working model: (1) The components of a functional RTC including the viral NSPs and host proteins are recruited, and the synthesis of −RNA begins at the 3′ end of gRNA; (2) The extension of new synthesis −RNA continues until the first functional TRS motif is encountered. Till now, there are two choices: (3) Ignore the existence of TRS and continue to synthesize full-length −RNA; or (4) Stop synthesizing and switch the template to TRS-L to complete −sgRNAs synthesis. The template is switched by the complementary binding between the 3′ end of the new synthesis −RNA and the TRS-L motif in the 5′ gRNA and the new −sgRNAs in turn acts as a template for the synthesis of sg mRNAs.

The synthesis of sg mRNAs by discontinuous transcription mechanism and the consequent 5′-3′ co-terminal nested sg mRNAs are distinctive characteristics of the coronavirus and arterivirus (Makino et al., 1988; Jeong and Makino, 1994; van Marle et al., 1999; Miller and Koev, 2000; van Vliet et al., 2002; Mateos-Gomez et al., 2013; Sola et al., 2015). Similar to genome replication, the sg mRNAs synthesis also proceeds within the DMVs. However, this process is more complex, conserved only in some members of *Nidovirales* (coronavirus, bafinivirus, and arterivirus), but not in others (okavirus). The discovery of multiple −sgRNA intermediates in cells infected with TGEV or MHV suggests that the process of negative-strand synthesis is discontinuous (Sawicki and Sawicki, 1990) (Figure 5B). Increasing evidences support the discontinuous transcription mechanism during the synthesis of −sgRNA intermediates plays a role in the generation of sg mRNAs within coronavirus and arterivirus (Sawicki and Sawicki, 1995; Sawicki et al., 2007). This model includes two central principles: (1) discontinuous transcription of −sgRNA intermediates; (2) the process of discontinuous transcription is similar to the mechanism of similarity-assisted or high-frequency copy-choice RNA recombination. The particular mechanical process can be viewed as an event that occurs continuously: (1) the synthesis of −RNA intermediates is facilitated by RTC at the 3′ end of the genome; (2) the extension of newly synthesized −RNA continues until the first functional transcription regulatory sequence (TRS-B) motif is encountered; RTC has two choices: (3) ignore the existence of TRS-B and continue to synthesize until encountering the next TRS-B, or continue to synthesize full-length −RNA intermediates; or (4) stop synthesizing and switch the template to 5′-leader sequence, with homologous leader transcription regulation sequence (TRS-L) to continue the synthesis. The mechanism of template switching is initiated by the complementarity between the 3′-end TRS-B on the newly synthesized −RNA and the TRS-L motif on the gRNA (Figure 5). The new −sgRNA intermediates would act as a template for synthesis of sg mRNAs (Sawicki and Sawicki, 2005; Pasternak et al., 2006). Transfection of *in vitro* synthesized sg mRNAs into cells suggests that long sg mRNAs containing multiple TRSs can also function as templates for synthesizing −sgRNA intermediates (Wu and Brian, 2010).

However, research findings on torovirus EToV and GAV have indicated that not all nidoviruses produces sg mRNAs with a common 5′ leader sequence and 3′ co-terminus (Pasternak et al., 2006). The EToV produces 4 sg mRNAs with 3′ co-terminus. Among these sg mRNAs, only the longest sg mRNA 2 (S gene) carries an 18-nt leader sequence derived from 5′ end of the virus genome via discontinuous RNA synthesis (Smits et al., 2005). In this case, a TRS is absent; fusion of non-continuous sequences appears to be regulated by a specific sequence element, which consists of a hairpin structure and 23-nt 3′ flanking stretches with sequence similar to a region located at the 5′ end of the genome. During the synthesis of −RNA intermediates, it is believed that the presence of hairpin structure can cause the transcriptase complex to detach, and then trigger a template switching mechanism similar to what occurs in arterivirus and coronavirus. The mRNA 3 (M), 4 (HE) and 5 (N) do not process a common 5′ leader sequence, but are fully colinear with the viral genome at 3′ end. They are proceeded by short noncoding regions called “intergenic,” which contain the conserved motif with a sequence pattern of 5′-ACN_3-4_CUUUAGA-3′. Representing the torovirus TRS equivalent, the motif does not act as sites for homology-assisted template-switching. Instead, it acts as terminators of transcription during the synthesis of −sgRNAs, and also play as promoters during the synthesis of sg mRNAs (van Vliet et al., 2002; Smits et al., 2005; Stewart et al., 2018). These findings suggest that EToV utilizes both discontinuous and non-discontinuous RNA synthesis mechanisms to generate its sg mRNAs.

The okavirus (GAV and YHV) produces three mRNA: gRNA1, sg mRNA2, and sg mRNA3. These RNAs are all co-terminal at 3′ end, and each possesses a 5′ cap structure and poly (A) tail (Sittidilokratna et al., 2008; Wijegoonawardane et al., 2008). mRNA2 and mRNA3 do not possess a common 5′-leader sequence. Similar to toronavirus mRNA 3 to 5, the okavirus mRNA2 and mRNA3, containing a 5′-GGUCAAUAVAAGGUA-3′ in the intergenic regions (IGRs) preceding gene 2 and gene 3, are produced by a “continuous” transcription strategy (Cowley et al., 2012). In this process, the IGRs serve as a dual function in the genome: whereas, they act as terminators during the synthesis of −RNA intermediates and as the transcriptional promotors during the production of sg mRNAs.

The presence of the common 5′ leader sequence in the −sgRNA intermediates may provide a conserved starting sequence for gRNA and all sg mRNAs synthesis; meanwhile, it may act as a recognition signal for the viral mRNA capping machinery, though no detailed study has been done so far; furthermore, it may enable viral mRNA to escape from virus-induced translation shut off, leading to the translation of viral mRNA as well as the impairment of host gene expression (Banerjee et al., 2020).

## 6 Regulation of RNA synthesis

Due to the intricate nature of the nidovirus RNA replication and transcription, the study on factors regulating RNA synthesis is still on the early stage compared to knowledge available for some other +RNA viruses. Here, we will provide a summary of the current knowledge regarding the viral and host factors involved in regulating viral RNA synthesis.

### 6.1 Regulation of RNA synthesis by RNA sequence

Synthesis of nidovirus RNA involves replication of full-length RNA and transcription of sg mRNAs. Many factors are involved in regulating RNA synthesis, with the first being the RNA sequence within the virus genome. These sequences are mainly located at the 5’-UTR and 3’-UTR of the genome or near the upstream of the encoding gene. The minimal sequences essential for TGEV, MHV, and IBV replication have been defined，which include a range of nucleotides located at the 5′ end (466 to 649 nt), at the 3′ end (388 to 493 nt), and at a poly (A) tail. These regions contain secondary and higher-order structures, known as cis-acting RNA elements which interacts with RNA motifs or replicases to initiate the RNA synthesis (Mukhopadhyay et al., 2009; Chen and Olsthoorn, 2010; Madhugiri et al., 2014).

**Figure 6:**
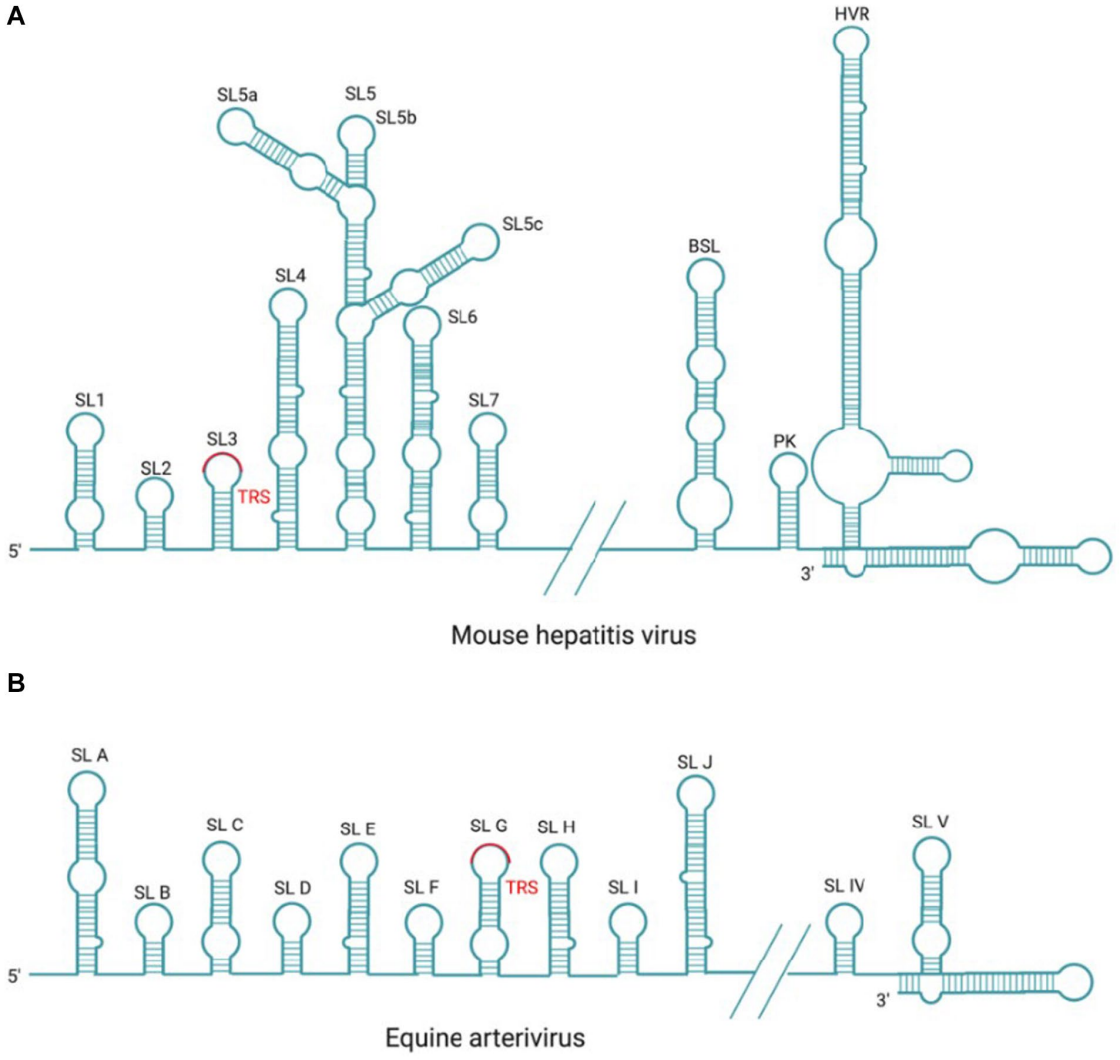
Cis-acting RNA element in MHV and EAV. **(A)** There are seven SL structures located in the 5′-UTR and three SL structures of BSL, PK, HVR located in the 3′-UTR within the coronavirus prototype MHV genome. **(B)** There are 10 SL structures located in the 5′-UTR and two SL structures located in the 3′-UTR within the arterivirus prototype EAV genome.

In coronavirus, the cis-acting RNA elements have a relatively conserved stem-loop (SL) structures (Raman et al., 2003; Kang et al., 2006; Li et al., 2008; Chen and Olsthoorn, 2010; Madhugiri et al., 2014; Figure 6A). Previous studies have demonstrated that SL structures plays a crucial role in the RNA synthesis through either long-distance RNA–RNA or RNA-protein interactions (Li et al., 2008; Yang et al., 2015). The SL structures at the 5′ end were first identified in BCoV, denoted from SL1 to SL6, respectively (Brown et al., 2007; Yang et al., 2015); however, there are seven SLs (SL1–SL7) in MHV (Guan et al., 2011). Among them, the SL1-SL4 are mapped within the 5′-UTR, while the rest SL structures are mapped into an ORF1a coding sequence. For BCoV, the SL5 and SL6 are located at the 5′-terminal 186 nt of NSP1 coding region (Brown et al., 2007). Compared to the two coronaviruses mentioned above, SARS-CoV-2 has one additional SL8 located at the 5′-end of the genome (Alhatlani, 2020). The number of SLs may vary with different coronaviruses, but SL1 to SL2 are conserved among all coronaviruses (Chen and Olsthoorn, 2010; Madhugiri et al., 2014). The SL1 is divided into two parts: the upper and the lower part, with the upper part participating in the coronavirus replication through base pairing. There is a dynamic model proposed for SL1 to mediate the interaction between 5′-UTR and 3′-UTR, thereby promoting the synthesis of −sgRNA intermediates (Li et al., 2008). SL2 is the most conserved 5’-UTR cis-acting RNA element that adopts a YNMG-type or CUYG-type tetraloop conformation (Liu et al., 2009; Lee et al., 2011), and mutation analysis has shown that it is essential for the sgRNAs synthesis (Liu et al., 2007). The leader core sequence (CS-L) and TRS-L, located within the SL3 or SL2, act as receptors for the nascent −RNA during discontinuous transcription (Sola et al., 2011). The conserved TRS-L plays a crucial role in regulating sg mRNAs transcription, which will be detailed in the subsequent TRS section. SL4, located downstream of SL3, is a long hairpin structure that probably functions as a spacer element in controlling the orientation of upstream SLs and TRS, and plays a role in directing sg mRNAs transcription (Yang et al., 2011; Vögele et al., 2021). In *α-Coronavirus*, SL5 is a higher-order structure with three hairpins (SL5a, SL5b, and SL5c) that extends into ORF1a (Chen and Olsthoorn, 2010) and it is also partially conserved in *β-Coronavirus*. Whereas, in IBV, SL5 is predicted to adopt a rod-like structure (Dalton et al., 2001). The structure phylogenetic analysis indicates that SL5 may help RNA interact with N protein and participates in genome packaging, as evidenced by study on TGEV (Morales et al., 2013). SL6 and SL7 are not necessary for coronavirus replication, and their role in RNA synthesis need to be further investigated (Yang et al., 2015).

There are three higher-order structures identified at the 3′-UTR as cis-acting RNA elements, which have been extensively studied in the MHV and BCoV. There are two specific RNA structures downstream of the N gene stop codon, the structurally and functionally conserved bulged stem-loop (BSL) of 68 nt and the hairpin RNA pseudoknot (PK) that consists of 54 nt and overlaps with the BSL of 5 nt. It has been reported that MHV PK loop1 directly interact with the 3′-end of the genome，and also with NSP8 and NSP9 (Zust et al., 2008). In most *β-Coronavirus*, BSL and PK are conserved and play crucial roles in viral RNA synthesis (Stammler et al., 2011; Zhao et al., 2020); however, in SARS-CoV-2 and *γ-coronavirus* IBV, PK is not observed (Dalton et al., 2001; Zhao et al., 2020). Downstream of the PK, there is a hypervariable region (HVR) that is highly divergent in sequence and structure among coronaviruses, but contains a conserved octa-nucleotide sequence 5′-GGAAGAGG-3′ (Goebel et al., 2007). In MHV, the HVR forms multiple SL structures in the last 160 nt of the viral genome, which is not essential in genome replication but affects pathogenicity *in vivo* (Goebel et al., 2007; Zust et al., 2008). Finally, the poly(A) tail functions as a cis-replication signal via interaction with PABPC1, which has been demonstrated in BCoV and MHV (Spagnolo and Hogue, 2000). BCoV and MHV-A59 defective interfering (DI) RNAs with truncated poly (A) tail consisting of 5A or 10 A residues were replicated at delayed kinetics, as compared to (DI) RNAs with wild-type poly (A) tail (>50 A residues; Spagnolo and Hogue, 2000, 2001).

In arterivirus, the cis-acting RNA elements at 5′-UTR and 3′-UTR also play essential roles in the replication and transcription of viral RNA. In EAV and PRRSV, the 5’-UTR cis-acting RNA elements include SLA, SLB, SLC, SLD, SLE, SLF, SLG, SLH, SLI, and SLJ (Figure 6B). SLG contains a conserved TRS-L, which is capable of base-pairing with TRS-B, and this interaction is essential for the −sgRNA intermediates synthesis (Pasternak et al., 2003; van den Born et al., 2005). Mutation analysis in SLB has indicated that the stem of SLB is essential for the synthesis of sg mRNAs during PRRSV infection (Lu et al., 2011); The 3′-UTR cis-acting RNA elements mainly include two putative hairpin structures (SLIV and SLV) and the base-pairing interaction between these two structures plays a role in the synthesis of viral genome and sg mRNAs (Verheije et al., 2001, 2002; Figure 6B). Mutation analysis in SLB has indicated that the stem of SLB is essential for the synthesis of sg mRNAs during PRRSV infection (Lu et al., 2011).

### 6.2 Transcription regulation sequence (TRS)

The TRS is an important cis-acting RNA element that plays a role in the transcription of nidovirus RNA. Hereby, the role of TRS in regulating RNA synthesis is summarized as following. Previous studies have found that the sg mRNAs of coronavirus, arterivirus, and bafinivirus carry a short 5′ leader sequence of 55–92 nt, 170–210 nt, and 42 nt, respectively (King et al., 2012), which is present at the 5′ end of the genome. This suggests that the sg mRNAs are synthesized by fusing non-contiguous sequence: the leader sequence at the 5′ end of the genome and the 5′ end of each gene coding sequence. Base-pairing is a key step during this non-contiguous fusion transcription process, which has been primarily demonstrated in arterivirus (van Marle et al., 1999; Pasternak et al., 2001) and coronavirus (Zúñiga et al., 2004).

Typically, two specific sequences called TRS-L and TRS-B, located at the 5′ end and proceeding upstream of each gene respectively, are responsible for the base pairing during sg RNA synthesis (Sola et al., 2005; Madhugiri et al., 2016). The TRS in coronavirus and arterivirus sg mRNAs were initially identified by sequencing the junction regions between the leader and body sequences of the sg mRNAs. The conserved sequence of the coronavirus TRS-L and TRS-B is about 7–18 nt, while the corresponding arterivirus TRS is usually about 5–8 nt (Gorbalenya et al., 2006), which is AU-rich. In toronavirus, there is a conserved 12 nt sequence element located upstream of ORF3, ORF4, and ORF5 (Di et al., 2018).

The TRS contains a conserved core sequence (CS) that is typically 6–7 nt in length and several flanking sequences. TRS-L includes the CS-L, while TRS-B includes the CS-B (Sola et al., 2011) (Figure 5A). Since the CS-L downstream of the 5′ leader sequence and all CS-B upstream of each body gene are identical in sequence, the CS-L can be paired with the complementary CS-B base of the newly synthesized −RNA, thus achieving the leader-body connection (Alonso et al., 2002; Sola et al., 2005). The CS-L and CS-B of the *Coronaviridae* and *Arterviridae* are list in Table 2.

**Table 2:** The CS-L and CS-B of the *Coronaviridae* and *Arterviridae.*

Family	Viruses	TRS		References
		CS-L	CS-B	
*Coronaviridae*	TGEV	5′-CUAAAC-3′	5′-GUUUAG-3′	Mateos-Gómez et al. (2011)
	PEDV	5′-AACGTAAA-3′	5′-UUUACGUU-3′	Yang et al. (2021)
	MERS-CoV	5′-AACGAAC-3′	5′-GUUCGUU-3′	Predicted
	HCoV-229E	5′-AACTAAAC-3′	5′-GUUUSGUU-3′	Predicted
	SARS-CoV	5′-ACGAAC-3′	5′-GUUCGU-3′	Thiel et al. (2003) and Hussain et al. (2005)
	SARS-CoV-2	5′-AACGAAC-3′	5′-GUUCGUU-3′	Wang D. et al. (2021)
	IBV	5′-CUUAACAA-3′	5′-UUGUUAAG-3′	Bentley et al. (2013)
	PDCoV	5′-ACACCA-3′	5′-UGGUGU-3′	Fang et al. (2016)
	EToV	5′-CUUUAGA-3′	5′-UVUAAAG-3′	Stewart et al. (2018)
	BCoV	5′-UCUAAA-3′	5′-UUUAGA-3′	Chang et al. (1996)
*Arteriviridae*	PRRSV	5′-UUAACC-3′	5′-GGUUAA-3′	van den Born et al. (2005) and Sola et al. (2015)
	EAV	5′-UCAACC-3′	5′-GGUUGA-3′	Pasternak et al. (2003)
	SHFV	5′-UCCUUAACC-3′	5′-GGUUAAGGA-3′	Di et al. (2017)

The discontinuous RNA transcription occurs during the synthesis of −sg RNA intermediates. Template switching is an important process during the transcription of sg RNA by RdRp, which needs the base-paring of TRS-L and TRS-B (Posthuma et al., 2017). When encountering the first TRS-B, RdRp stops the synthesis along with the original template and switches to the 5′-leader sequence with homologous TRS-L to complete its synthesis (Sola et al., 2015). This long-distance RNA–RNA interaction promotes the synthesis of −sgRNA intermediates, which in turn serves as a template for sg mRNAs synthesis (Mateos-Gomez et al., 2013).

### 6.3 Regulation of RNA synthesis by cellular proteins and viral proteins

The factors involved in viral RNA synthesis were identified by studying their binding to viral genome or replicase proteins (Sawicki et al., 2005; Shi and Lai, 2005). Several cellular and viral proteins have been identified to be involved in regulation of nidovirus RNA replication and transcription (van Vliet et al., 2002; Pasternak et al., 2006; Ulferts and Ziebuhr, 2011; Posthuma et al., 2017; de Wilde et al., 2018; Yan et al., 2020). These factors mainly regulate the formation of RTC and the binding of RTC to cis-acting RNA elements through protein-RNA and protein–protein interactions. These host and viral proteins are as well as their interaction proteins are summarized in Table 3.

**Table 3:** Summary of host and viral proteins regulating RNA synthesis.

Family	Protein		Interaction proteins	References
*Coronaviridae*	Host	hnRNPA1	TRS, PTB, N protein,	Stohlman et al. (1988), Zhang and Lai (1995), Li et al. (1999), Wang and Zhang (1999), Shi et al. (2000), and Huang and Lai (2001)
		PTB (hnRNP1)	TRS, hnRNPA1, N protein	Huang and Lai (1999, 2001) and Choi et al. (2002)
		PABP	Poly (A) tail	Spagnolo and Hogue (2000)
		DDX	N protein	Wu et al. (2014)
	Virus	NSP1	NA	Molenkamp et al. (2000), Tijms et al. (2001), Tijms et al. (2007), Sun et al. (2009), and Nedialkova et al. (2010)
		NSP3	NSP4, NSP6	Angelini et al. (2013) and Oudshoorn et al. (2017)
		NSP4	NSP3, NSP6	Angelini et al. (2013) and Oudshoorn et al. (2017)
		NSP9	Single-stranded RNA, NSP12	Egloff et al. (2004), Sutton et al. (2004), Ponnusamy et al. (2008), Snijder et al. (2016), and Yan et al. (2021)
		NSP6	NSP3, NSP4	Angelini et al. (2013) and Oudshoorn et al. (2017)
		NSP12	NSP5, NSP8, NSP9	Brockway et al. (2003)
		NSP13	DDX15	Chen J.-Y. et al. (2009)
		N protein	DDX1, TRS, Poly (A)	Grossoehme et al. (2009), Wu et al. (2014), Snijder et al. (2016), Tsai et al. (2018), and Yan et al. (2021)
*Arteriviridae*	Host	Cycliphilin	NSP5	de Wilde et al. (2013) and de Wilde et al. (2019)
		Cyclin-dependent kinase 9	NA	Wang M. D. et al. (2021)
		DHX9	N protein, NSP9	Liu et al. (2016)
		Nucleotide-binding oligomerization domain-like receptor (NLR) X1	NSP9	Jing et al. (2019)
		poly(C) binding protein (PCBP)	NSP1β	Napthine et al. (2016)
	Virus	N protein	DHX9	Liu et al. (2016)
		NSP1	P100	Tijms and Snijder (2003)
		NSP2	NSP3	Snijder et al. (2001)
		NSP3	NSP2	Snijder et al. (2001)
		NSP5	NSP2, NSP3	Snijder et al. (2013)
		NSP9	DHX9, NLRX1,	Liu et al. (2016)
		NSP10	NA	Seybert et al. (2000b) and Lehmann et al. (2015c)
		NSP12	NSP11	Song et al. (2018)

#### 6.3.1 Protein-RNA interaction

The protein-RNA interaction regulates the RNA synthesis process (Sola et al., 2011). It has been identified that the viral and cellular factors bind to RNA genome or replicases so as to drive the RNA synthesis (Sawicki et al., 2005; Shi and Lai, 2005; Galan et al., 2009; Xu et al., 2010). The common strategies employed to identify the replicase components are: genome wide two-hybrid screening, proteomic analysis, high-throughput functional assay using host cell mutants or siRNA, *in vitro* translation or transcription systems.

Most of the NSPs encoded by ORF1a and ORF1ab, together with N protein and cellular proteins, form the membrane-associated RTC. This complex interacts with viral RNA, and plays a crucial role in mediating the synthesis of viral genome and sg mRNAs. For coronavirus, the enzymes involved in RNA synthesis are the NSP7-NSP8 primase complex, NSP9 dimers, potential molecular switch (NSP10), RdRp (NSP12), helicase (NSP13), ExoN (NSP14), EndoU (NSP15), MTase (NSP16), and some unidentified cellular proteins. Among them, RdRp, helicase, and N protein are essential components for RTC；other NSPs and cellular proteins also contribute to the formation of RTC and regulation of RNA synthesis. The role of the replicases encoded by ORF1a and ORF1b in the formation of RTC has been reviewed in section 4 and 5. Hereby, we will focus on how N protein and cellular proteins interact with viral RNA and play a role in the synthesis of viral RNA.

##### 6.3.1.1 N protein

The N protein serves as structural protein within the virion and plays a crucial role in viral transcription and replication. It forms oligomers and binds to gRNA, resulting the formation of helical ribonucleoprotein complexes by wrapping gRNA (Chang et al., 2006; Chen et al., 2007; Lo et al., 2013; Cong et al., 2017; Gui et al., 2017). These complexes are then incorporated into viral particles by interaction with the C-terminus of M proteins (Kuo et al., 2016). In addition to protecting gRNA, N protein regulates the replication and transcription of viral RNA, acting as a chaperone to promote gRNA replication (Almazán et al., 2004; Schelle et al., 2005; Zúñiga et al., 2010). The interaction with NSP3 enables N protein to be recruited to DMVs and associated with RTC (Hurst et al., 2010, 2013; Keane and Giedroc, 2013; Tatar and Tok, 2016; Cong et al., 2020). N protein might be involved in the discontinuous transcription of sg mRNAs, as depletion of N from the replicon reduces the production of sg mRNAs rather than gRNA (Zúñiga et al., 2010). In SARS-CoV-1, the N-terminal domain of N protein binds specifically to TRS-L sequence and enhances the unwinding of TRS duplexes (Grossoehme et al., 2009). In addition, N protein possesses RNA chaperone activity to facilitate the template switching, which is essential for efficient transcription of −sgRNA intermediates (Almazán et al., 2004; Zúñiga et al., 2010). The serine and arginine (SR) rich region, which links N-terminus and C-terminus of N protein, is modified by phosphorylation (Peng et al., 2008; Wu et al., 2009), which results in the differentiation between the binding of viral RNA and cellular mRNA (Chen et al., 2005; Spencer et al., 2008). In IBV, phosphorylation of N protein by GSK-3 also enables the recruitment of cellular RNA helicase DDX1 to RTC, which in turn enables the continuous synthesis of longer sg mRNAs and gRNA by promoting template read-through and transition from discontinuous transcription (Wu et al., 2009, 2014). This mechanism guarantees a proper balance among the synthesis of gRNA, long sg mRNAs, and short sg mRNAs. GSK-3 has been proved to be essential for the phosphorylation of SARS-CoV-2 N protein and the synthesis of viral RNA, serving as a promising target for developing pharmaceuticals to treat COVID-19 (Wu et al., 2014; Liu et al., 2021). A non-redundant dataset containing 495 compounds for GSK3α and 3,070 compounds for GSK3β has been applied to virtual high-throughput screening and two drugs (selinexor and ruboxistaurin) have been selected for further investigation (Pirzada et al., 2023). Therefore, interference with the phosphorylation of N protein by targeting GSK-3 is a feasible strategy to combat against the coronavirus associated diseases.

##### 6.3.1.2 Cellular proteins

Cellular proteins perform their function in viral RNA synthesis via binding to 5′-UTR, internal TRS-B, 3′-UTR, or RTC. The proteins binding to 5′-UTR or 3′-UTR probably participate in viral RNA replication, transcription, translation and stability, while the proteins binding to TRS-B might help the discontinuous transcription.

There are two cellular heterogeneous nuclear ribonucleoproteins, polypyrimidine-tract binding protein (PTB) and hnRNPA1, which participate in the RNA transcription. PTB protein, also known as hnRNP I, plays an important role in regulating the alternative splicing of pre-mRNAs and translating the mRNA (Kaminski et al., 1995; Svitkin et al., 1996; Valcarcel and Gebauer, 1997). During MHV infection, PTB binds to the TRS-L (with UCUAA pentanucleotide repeats) which is located in the 5′-UTR (56–112 nt; Li et al., 1999). Deletion of these leader sequences in DI RNAs results in the reduced RNA transcription, indicating that the binding of PTB to TRS-L might regulate the transcription process. Another study has also demonstrated that PTB binds to the TRS-L sequence of TGEV genome, as identified by the RNA-protein pull-down assay and proteomic analysis (Galan et al., 2009). These findings suggest this protein might play a general role in coronavirus RNA transcription. Interestingly, PTB also interacts with the complementary strand of the 3′-UTR (c3′-UTR), with a strong binding site between 53 and 149 nt and a weak binding site between 270 and 307 nt on the c3′-UTR (Huang and Lai, 1999). The binding of PTB to 53–149 nt leads to a conformational change in the neighboring RNA region. When partial deletion occurs within the PTB-binding sequence, it completely abolishes the conformational change induced by PTB, and impairs the ability of the −RNA to transcribe mRNAs (Huang and Lai, 1999). Thus, the binding of PTB to c3’-UTR may play a crucial role in mRNA transcription by changing the c3′-UTR conformation. PTB has been found to stimulate the internal ribosome entry site (IRES) mediated translation, by interacting with picornavirus IRES elements (Kaminski et al., 1995; Niepmann, 1996; Niepmann et al., 1997). It may exert influence on the IRES-mediated translation of MHV 5b, and IBV 3c, which are ORFs that encode the envelope protein (Thiel and Siddell, 1994; Lai and Cavanagh, 1997; Jendrach et al., 1999). Meanwhile, the interaction between PTB with N protein suggests a potential contribution to the formation of RNP complex (Choi et al., 2002). In summary, according to research findings, the interaction between PTB and MHV RNA leader sequence or sequence complementary to the 3’-UTR is involved in RNA transcription, and the interaction between N protein and PTB also modulates transcription.

As is widely known, another cellular heterogeneous nuclear ribonucleoprotein, hnRNPA1 facilitates the pre-mRNA splicing and transport of cellular RNAs in the nucleus (Dreyfuss et al., 1993), as well as modulates the mRNA translation and turnover in the cytoplasm (Hamilton et al., 1993, 1997; Svitkin et al., 1996). HnRNPA1 was initially found to specifically bind to MHV cTRS-L and cTRS-B present in the −RNA (Furuya and Lai, 1993; Li et al., 1997), which suggests that HnRNPA1 is important for the discontinuous viral RNA transcription. It has been found that mutagenesis of the TRS-B in the DI RNA system will lead to reduced transcription, which was correlated with relative binding affinity of the cTRS-B sequence to hnRNPA1 (Furuya and Lai, 1993; Li et al., 1997; Huang and Lai, 2001). According to another study, a vital hnRNPA1-binding site has been identified within the HRV domain, which is located between 90 and 170 nt from the 3′ end of MHV RNA, while a weak binding site has been identified between 260 and 350 nt (overlapping with the BSL) from the 3′ end (Huang and Lai, 2001). Overexpression of hnRNPA1 results in an acceleration of MHV RNA synthesis, and the expression of dominant-negative hnRNPA1 mutant leads to a global inhibition of viral RNA (Dalton et al., 2001). Additionally, hnRNPA1 interacts with N protein of MHV and forms a component of the RTC (Wang and Zhang, 1999). It also plays a role in facilitating the formation of the RNP complex via binding to the negative-stranded cTRS-L and cTRS-B (Zhang et al., 1999). The extent of hnRNPA1 binding to the cTRS is directly related to the transcription efficiency in the MHV model (Zhang and Lai, 1995). Meanwhile, it has been observed that the hnRNPA1 interacts with the 3′ end of the genome in the TGEV (Luo et al., 2005). In another study, it has been found that hnRNPA1 interacts with N protein of PEDV, and the silenced expression of hnRNPA1 impairs viral replication. The interaction between hnRNPA1 and N protein has also been found in SARS-CoV (Luo et al., 2005), SARS-CoV-2 (Perdikari et al., 2020), IBV (Emmott et al., 2013). Whereas, in the hnRNPA1 defective mouse erythroleukemia cell line CB3 (Ben-David et al., 1992), it has been observed that efficient MHV replication still occurs (Shen and Masters, 2001). The interaction of hnRNP A/B, hnRNP A2/B1, and hnRNP A3 with the MHV negative-stranded leader RNA potentially substitutes for hnRNPA1 in regulating MHV RNA replication (Shi et al., 2003).

It is interesting to note that the hnRNPA1 binding sites on 3′ end of MHV genome are complementary to the sites on the −RNA intermediates that bind to PTB (Huang and Lai, 1999; Li et al., 1999; Huang and Lai, 2001). Mutations that affect PTB binding to the negative strand of the 3’-UTR also hinder hnRNPA1 binding on the positive strand, demonstrating that hnRNPA1 and PTB work together to mediate potential 5′-3′ cross talks in MHV RNA, which plays an important role in RNA replication and transcription.

HnRNP Q, also known as SYNCRIP, is capable of binding to the 5′-UTR or to the complementary sequence c5′-UTR of MHV (Choi et al., 2004). Meanwhile, it has been shown that hnRNP Q bind to TEGV 3′-end genome and positively regulates the synthesis of viral RNA (Galan et al., 2009). As elaborated in a recent study, another hnRNP family member, hnRNP C, is involved in promoting the replication of MERS-CoV and SARS-CoV-2 by regulating the expression of a specific subset of circRNAs and cognitive mRNAs (Zhang X. et al., 2022). The positive role of hnRNPs in viral RNA replication/transcription renders these proteins as broad-spectrum antiviral targets. A hnRNPA2B1 agonist has been demonstrated to effectively inhibit HBV and SARS-CoV-2 omicron *in vivo* (Zuo et al., 2023).

Poly (A)-binding protein (PABP) is a protein that binds to the 3′ poly (A) tail on eukaryotic mRNAs, with its main function being to promote both mRNA translation initiation and mRNA stability. For BCoV, MHV, and TGEV, PABP has been identified as binds to the 3’ UTR and poly (A) tail (Lin et al., 1994; Spagnolo and Hogue, 2000; Galan et al., 2009). It has been found that the binding of PABP to 3’-UTR of DI RNA replicons is associated with the replication of DI RNA (Yu and Leibowitz, 1995a,b; Liu et al., 1997; Huang and Lai, 2001). The interaction between PABP and poly (A) tail may have a direct role in coronavirus replication and transcription, which can mediate the interaction between the 5′ and 3′ ends of coronavirus RNA (Kim et al., 1993; Lin et al., 1994, 1996; Lai, 1998), or indirectly modulate the synthesis of viral RNA by affecting the translation process. Wang et al. have illustrated that PABPC1 interacts with the N protein of arterivirus PRRSV and involves in viral replication (Wang et al., 2012); whereas, Tsai et al. have found that the interplay among PABP, N protein and poly(A) tail mainly regulates coronavirus mRNA translation (Tsai et al., 2018).

Other cellular proteins associated with coronavirus RTC include the cellular DEAD box helicase family. This multifunctional protein family is involved in various steps of RNA life cycle, such as transcription, mRNA splicing, RNA transport, translation, RNA decay. The specific interaction between DDX5 and SARS-CoV NSP13 (helicase) is involved in viral RNA synthesis (Chen J.-Y. et al., 2009); and the interaction between DDX1 with IBV and SARS-CoV NSP14 also enhances virus replication (Xu et al., 2010). Interestingly, when the N protein is phosphorylated, it recruits the RNA helicase DDX1 to the phosphorylated-N-containing complex, which in turn facilitates the process of template readthrough and enables the synthesis of longer sg mRNAs; afterwards, the transition from discontinuous to continuous transcription guarantees the balance between sg mRNAs and full-length gRNA (Wu et al., 2014). The N protein of SARS-CoV-2 has been found to interact with several RNA helicases, including DDX1, DDX3, DDX5, DDX6, DDX21, and DDX10; among them, DDX1, DDX5, and DDX6 are essential for virus replication, while DDX21 and DDX10 restrict the viral infection (Ariumi, 2022). All the above studies reveal that the hijacking of host cellular DDX helicases for viral replication and transcription is a general strategy among coronaviruses.

During PRRSV infection, DDX18 redistributes from nucleus to cytoplasm and interacts with NSP2 and NSP10, to promote virus replication (Jin et al., 2017). DDX21 is also translocated from nucleus to cytoplasm and then positively regulates the PRRSV replication by stabilizing the expression of PRRSV NSP1α, NSP1β, and N protein (Li et al., 2022). It has been found that DDX21 interacts with NSP1β, which enhances the expression of DDX21. Another DDX family member, DDX5, has been found to interact with NSP9, the RdRp, thereby positively regulating the replication of PRRSV (Zhao et al., 2015). Moreover, PRRSV infection promotes the DDX10 to translocate from the nucleus to the cytoplasm for macroautophagic/autophagic degradation. Additionally, the viral E protein interacts with and promotes the selective autophagic degradation of DDX10, to antagonize the antiviral effect of this protein (Li et al., 2023).

#### 6.3.2 Protein–protein interaction

The NSP12 (RdRp), NSP13 (helicase), and N protein are crucial for the replication and transcription of coronavirus RNA. In addition, other viral proteins also contribute to the regulation of RNA synthesis. For example, NSP3, NSP4, and NSP6 are responsible for the formation of DMVs, while NSP3 and NSP5 have the activity to process pp1a and pp1ab so as to produce mature replicases. In MHV, NSP12 (RdRp) has been shown to interact with 3CLpro, NSP8, and NSP9 to perform the RNA synthesis (Brockway et al., 2003). It has been shown that NSP9 forms dimers and binds to single-stranded RNA in a non-sequence-specific manner (Egloff et al., 2004; Sutton et al., 2004; Ponnusamy et al., 2008). Recently, it has been shown that the N terminus of NSP9 inserts into the catalytic center of NiRAN domain of NSP12, which in turn inhibits the activity of NSP12 (Snijder et al., 2016; Yan et al., 2021).

In arterivirus, NSP9 (RdRp), NSP10 (helicase), and N protein play crucial roles in the processes of replication and transcription. NSP2, NSP3, and NSP5 contain TM responsible for remodeling intracellular membranes and recruiting other viral replicases to RTC (Snijder et al., 1994; van der Meer et al., 1998; Snijder et al., 2001; Posthuma et al., 2008). The ability of arterivirus RTC to synthesize RNA *in vitro* is dependent on a host factor (van Hemert et al., 2008).

As a multifunctional protein during EAV infection that contains two papain-like cysteine protease (PCPα and PCPβ) and a zinc-finger motif, NSP1 plays an important role in regulating the viral RNA synthesis and virion biogenesis, as well as in controlling the balance between genome replication and sg mRNAs synthesis (Molenkamp et al., 2000; Tijms et al., 2001, 2007; Nedialkova et al., 2010). In addition, the Zinc-finger motif, located in the N-terminal region of PRRSV NSP1, was involved in regulating sg RNA synthesis (Sun et al., 2009). Therefore, NSP1 protein in arterivirus is a multifunctional protein involved in proteolytic maturation of the replicase and the regulation of RNA transcription.

Paraoxonase-1(PON1), an esterase with specifical paraoxonase activity, interacts with PRRSV RdRp (NSP9) and plays a role in facilitating the NSP9 function in PRRSV replication; moreover, it has been proved to reduce the type I IFN signaling during PRRSV infection (Zhang L. et al., 2022). RBM39, a nuclear protein involved in transcriptional activation and precursor mRNA splicing, relocates from nucleus to cytoplasm to bind with viral RNA, thereby prompting the PRRSV replication (Song et al., 2021).

## 7 Conclusion

Since the first in-depth analysis on the replication and transcription of *Nidovirales* in 1980 (Evans and Simpson, 1980), significant progress has been made in the understanding of mechanisms and regulation of sg mRNAs generation, and the accurate synthesis mechanism has been determined. During nidovirus infection, a nested set of sg mRNAs were produced, all sharing a common 5′ leader sequence and 3′ co-terminus. It has been demonstrated that the nested −sgRNA intermediates are produced through the discontinuous synthesis mechanism from the gRNA template. Afterwards, these −sgRNA intermediates serve as templates for the synthesis of sg mRNAs. In this process, viral and cellular factors are known to form the RTC and regulate the activities of replicases, such as the N protein, replicases, host cellular hnRNP, PTB, and DDX. In addition, the interactions among RNA–RNA, protein-RNA, and protein–protein play a role in regulating the replication and transcription. The viral transcription and replication machinery represents an attractive target for developing antiviral drugs. For example, the lead compounds, remdesivir and nirmatrelvir, specifically targeting at SARS-CoV-2 RdRp and Mpro respectively, have already been approved for COVID-19 treatment (Gao et al., 2020; Lamb, 2020; Lee et al., 2022; Amani and Amani, 2023; Harris, 2023). Thus, detailed insights provide new opportunities for designing structure-based antiviral drugs, which target at multiple aspects of the RNA synthesis processes.

## Author contributions

YL: Conceptualization, Project administration, Supervision, Writing – original draft, Writing – review & editing, Formal analysis, Funding acquisition. HW: Conceptualization, Writing – original draft. HL: Writing – review & editing, Formal analysis. YS: Formal analysis, Supervision, Writing – review & editing. LT: Supervision, Writing – review & editing. CS: Project administration, Writing – review & editing. XQ: Supervision, Writing – review & editing. CD: Funding acquisition, Supervision, Writing – review & editing.
